# Rootstock–scion exchanging mRNAs participate in the pathways of amino acid and fatty acid metabolism in cucumber under early chilling stress

**DOI:** 10.1093/hr/uhac031

**Published:** 2022-02-19

**Authors:** Wenqian Liu, Qing Wang, Ruoyan Zhang, Mengshuang Liu, Cuicui Wang, Zixi Liu, Chenggang Xiang, Xiaohong Lu, Xiaojing Zhang, Xiaojun Li, Tao Wang, Lihong Gao, Wenna Zhang

**Affiliations:** 1 Beijing Key Laboratory of Growth and Developmental Regulation for Protected Vegetable Crops, China Agricultural University, Beijing 100193, China; 2 College of Life Science and Technology, HongHe University, Mengzi, Yunnan 661100, China

## Abstract

Cucumber (*Cucumis sativus* L.) often experiences chilling stress that limits its growth and productivity. Grafting is widely used to improve abiotic stress resistance by exploiting a vigorous root system, suggesting there exists systemic signals communication between distant organs. mRNAs are reported to be evolving fortification strategies involving long-distance signaling when plants suffer from chilling stress. However, the potential function of mobile mRNAs in alleviating chilling stress in grafted cucumber is still unknown. Here, the physiological changes, mobile mRNA profiles, and transcriptomic and metabolomic changes in above- and underground tissues of all graft combinations of cucumber and pumpkin responding to chilling stress were established and analyzed comprehensively. The co-relationship between the cluster of chilling-induced pumpkin mobile mRNAs with differentially expressed genes and differentially intensive metabolites revealed that four key chilling-induced pumpkin mobile mRNAs were highly related to glycine, serine, and threonine synthesis and fatty acid β-oxidative degradation metabolism in cucumber tissues of heterografts. The verification of mobile mRNAs, potential transport of metabolites, and exogenous application of key metabolites of the glycerophospholipid metabolism pathway in cucumber seedlings confirmed the role of mobile mRNAs in regulating chilling responses in grafted cucumber. Our results build a link between the long-distance mRNAs of chilling-tolerant pumpkin and the fatty acid β-oxidative degradation metabolism of chilling-sensitive cucumber. They also help to uncover the mechanism of signaling interaction between scion and stock as a means of achieving chilling tolerance in grafted cucumber.

## Introduction

Cucumber (*Cucumis sativus* L.) is one of main vegetables produced via protected cultivation in China in winter. Due to the shallow root system, cucumber often suffers abiotic stress that can limit quality and productivity, particularly chilling stress (<10°C) [1]. Chilling stress is an environmental stress that can inhibit plant growth and development, such as leaf development inhibition, flower sterility, and changes in membrane lipid biology and enzyme conformation [2–6], ultimately causing plant death. Accordingly, a series of physiological, biochemical, and molecular mechanisms have been developed for adaptation to cold temperatures in plants [7–10].

Grafting is widely used to increase cucumber resistance and improve plant growth vigor to increase productivity in stress environments such as salt, alkalinity, drought, heat, and particularly chilling [11–19]. In addition to the well studied physiological responses of grafting in conferring chilling tolerance on horticultural crops [20–22], transcriptomic profiling also has provided considerable evidence that traditional rootstocks improve tolerance of chilling, autotoxin substances, and low-phosphate stress [23–25]. Moreover, metabolome profiling analysis, which is widely used to investigate important agricultural traits such as flavor, yield, biomass, and nutritional quality, confirmed that grafting significantly increases the contents of primary metabolites such as sugar, organic acids, and amino acids in cucumber [26] and watermelon fruits [27]. The most interesting phenomenon is that the acquisition of chilling tolerance in grafted crops depends on the tolerance capacity of both rootstock and scion, and the different chilling tolerances of different combinations of rootstocks and scions [28, 29]. Therefore, there exists signaling communication in response to chilling tolerance between scion and rootstock.

Along with water and minerals, systemic signals such as hormones, photosynthetic assimilation, amino acids, proteins, and RNAs participate in plant growth, cell division, and systemic acquired resistance via the plant’s vasculature system [18, 30–32]. More thorough research found that the participation of systemic mobile mRNAs such as *CmNACP*, *SHR*, *KNOTTED1*, *StBEL5*, *PFP-LET6*, and *CmGAI* in the development of roots, apical meristem, leaves, and underground tubers in various plant species involves their long-distance transport between organs, facilitated by companion cells and sieve elements of the phloem system, and constitutes an important aspect of non-cell-autonomous signaling in plants [33–37]; likewise, the movement of *CmWRKYP*, *CmPP2*, *Cmlec17*, *CmPP16*, and *SlSSI* mRNAs is involved in stress responses [38–42]. With bioinformatic tools, numerous mRNAs and non-coding RNAs have been shown to move from source into sink organs in response to chilling, phosphorus deficiency, and salt stress resistance by using grafting [23, 43–45]. Gene Ontology (GO) analyses have revealed a broad range of biological processes and molecular functions for mobile mRNAs [44, 46, 47]; however, little is known about how such mobile mRNAs, as rootstock–scion interactional signals, are involved in improving chilling tolerance in grafted cucumber. Therefore, there remains a need to explore the correlation between rootstock–scion mRNAs and physiological characteristics and metabolomic and transcriptomic changes in the response to chilling stress in grafted cucumber.

Thus, in this study, to elucidate the effect of grafting on cucumber chilling resistance, we firstly compared the morphological and physiological characteristics of all four possible homo- and heterografted cucumber/pumpkin combinations in response to different periods of chilling stress. To further clarify the metabolomic and transcriptional changes during the early chilling response, we carried out untargeted liquid chromatography–mass spectrometry (LC–MS) metabolomics profiling to identify differentially intensive metabolites (DIMs) and differentially expressed genes (DEGs) from the leaves and roots of all grafts under chilling stress. We then profiled chilling-induced mobile mRNAs and clustered the functional characteristics of these mRNAs. To explore the molecular mechanisms acting in the grafted cucurbit combinations during chilling, we carried out an integrated analysis of the profiles of chilling-induced mobile mRNAs, DEGs, and DIMs based on KEGG (Kyoto Encyclopedia of Genes and Genomes) pathways. Here, identifying the key functions and characterizing the pathways of a large number of mobile mRNAs under early chilling conditions yielded a dataset that provides new insights into physiological performance, in combination with transcriptional and metabolic influences that occur in heterologous organs in heterografts during chilling stress. Our results are thus an important step toward a fuller understanding of the mechanisms underlying the rootstock–scion interaction on grafted horticultural crop growth and stress resistance.

## Results

### Cucumber heterografts show tolerance of early chilling stress

**Figure 1:**
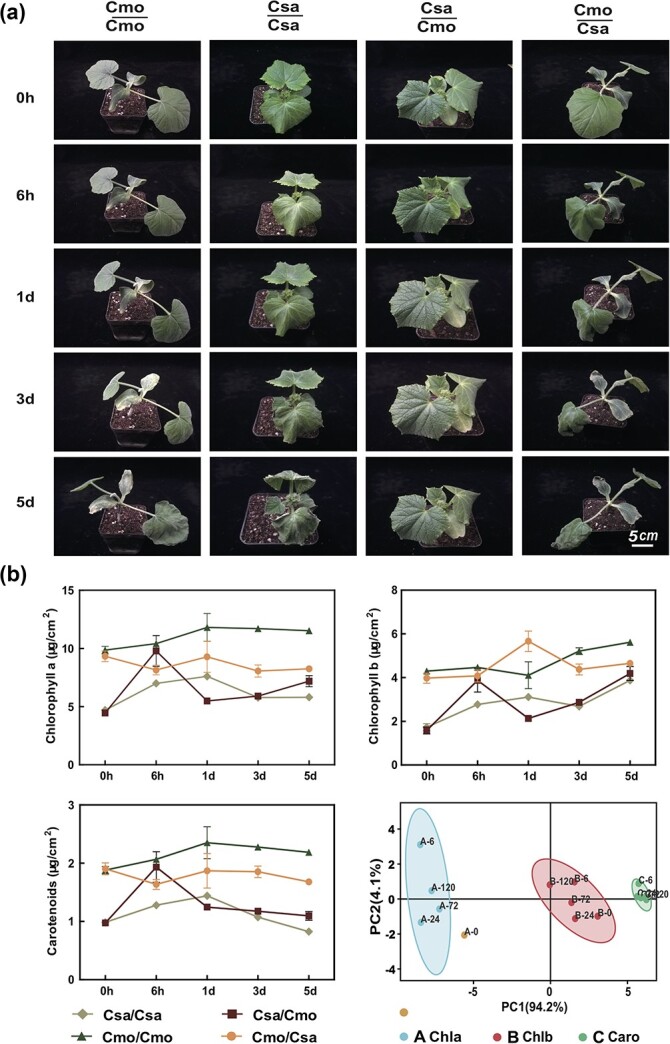
Grafting alleviates Csa chilling tolerance in physiological changes. **a** Phenotypic changes in all grafted combinations in a chilling-treatment time course. At least three biological replicates of all graft combinations at each time point were used for imaging. **b** Chlorophyll a (Chla), chlorophyll b (Chlb) and carotenoid (Caro) contents in the first leaf of homo- and heterografted seedlings after different periods of chilling stress. Three replicates of the pooled first true leaves and 9 or 10 individual seedlings per replicate at each time point were used for the analysis, and results from PCA of these data (bottom right). Error bars represent the standard deviation.

To understand early chilling tolerance, we assessed the status of homograft cucumber/cucumber (Csa/Csa) (scion/rootstock) and pumpkin/pumpkin (Cmo/Cmo) and heterograft cucumber/pumpkin (Csa/Cmo) and pumpkin/cucumber (Cmo/Csa) seedlings constructed by hypocotyl grafting and subjected to chilling stress at the two-true-leaf stage. The first true leaves of homografted Csa/Csa and Cmo/Cmo plants started to dehydrate after 6 and 12 hours of 4°C chilling treatment, respectively, whereas those of heterografted Cmo/Csa and Csa/Cmo plants began to dehydrate after 12 and 6 hours, respectively (Fig. 1a). To elucidate the physiological differences among all grafts during chilling responses, we analyzed the morphological and physiological characteristics of heterografted and homografted Csa after chilling stress versus those in the absence of chilling. Under control condition (0 days of chilling), the contents of chlorophyll a, chlorophyll b and carotenoid in all grafts followed the highest to lowest order of Cmo/Cmo, Cmo/Csa, Csa/Cmo, Csa/Csa. After 6 hours of chilling stress, the contents of chlorophyll a, chlorophyll b and carotenoid in the first leaves of Csa/Cmo and Csa/Csa grafts were significantly increased due to dehydration, but the chilling responses followed the highest to lowest order of Cmo/Cmo, Cmo/Csa,Csa/Cmo, Csa/Csa. After chilling treatment, the leaf contents of chlorophyll a and carotenoid were significantly higher in Cmo/Cmo than in Cmo/Csa. Chlorophyll b increased temporarily at 1 day before decreasing again in Cmo/Csa, but it remained higher in Cmo/Csa than in Cmo/Cmo (Fig. 1b). In Csa leaves, the chlorophyll a, chlorophyll b, and carotenoid contents of the first leaves of Csa/Cmo were dramatically increased after 6 hours of chilling and then decreased after 1 day of chilling compared with that of Csa/Csa, but on the 5th day the contents exhibited chilling tolerance following the highest to the lowest order of Cmo/Cmo, Cmo/Csa, Csa/Cmo, Csa/Csa (Fig. 1b).

To assess the chilling tolerance capacity of all graft combinations, we performed a principal components analysis (PCA) with multiple indices including the chlorophyll a, chlorophyll b, and carotenoid contents at 0 hours, 6 hours, 1 day, 3 days and 5 days of chilling treatment. The results indicated that principal components 1 and 2 (PC1 and PC2) accounted for 92.2 and 5.6% of the total physiological parameters (Supplementary Data Table S1). Based on PC1 data, by comparison with other indices the chlorophyll a content index at 6 hours was considered an indicator for evaluating plant chilling tolerance (Fig. 1b; Supplementary Data Table S1). Reverse transcription–quantitative PCR (RT–qRCR) results showed that, compared with homografts, the mRNA abundance of cold-responsive marker genes such as *CAMTA*s, *CBF3*, and *ICE*s was significantly induced in the leaf and root of heterografts at 6 hours (Supplementary Data Fig. S1). Therefore, to summarize, the chilling tolerance of all graft combinations can be evaluated by using the chlorophyll a content index at 6 hours of chilling treatment; i.e. the chilling tolerance performance of graft combinations followed the highest to lowest order of Cmo/Cmo, Csa/Cmo, Cmo/Csa, Csa/Csa.

### Metabolite profiles reveal differences in metabolic regulation of early chilling responses in different graft combinations

To elucidate the metabolic responses in all graft combinations in the 6-hour chilling condition, we performed untargeted LC–MS profiling with the first leaf and root of each grafting combination. To simplify discussion of the results, we use the abbreviations Normal_leaf^Cmo/Cmo^, Normal_leaf^Csa/Csa^, Normal_leaf^Csa/Cmo^, and Normal_leaf^Cmo/Csa^ to represent the first leaves of Cmo/Cmo, Csa/Csa, Csa/Cmo, and Cmo/Csa grafts in the normal-temperature growth condition; Normal_root^Cmo/Cmo^, Normal_root^Csa/Csa^, Normal_root^Csa/Cmo^, and Normal_root^Cmo/Csa^ for the roots of the respective grafts in the normal-temperature growth condition; Cold_leaf^Cmo/Cmo^, Cold_leaf^Csa/Csa^, Cold_leaf^Csa/Cmo^, and Cold_leaf^Cmo/Csa^ for their first leaves after 6 hours of chilling stress; and Cold_root^Cmo/Cmo^, Cold_root^Csa/Csa^, Cold_ root^Csa/Cmo^, and Cold_ root^Cmo/Csa^ for the roots after 6 hours of chilling stress. In total, from the leaves and roots of all graft combinations, we identified 900 negative compound and 1058 positive compound compounds (Supplementary Data Table S2).

**Figure 2:**
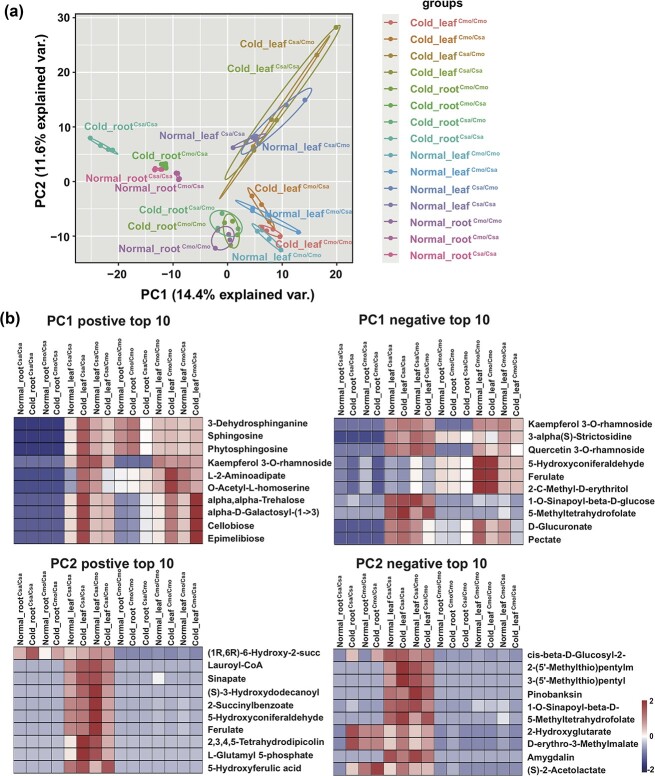
PCA of metabolite profiles in leaves and roots of all grafted combinations under control condition and 6 hours chilling stress. LC–MS measurements were performed on samples from leaves and roots of all graft combinations under control condition and after 6 hours of chilling. Four independent biological replicates (one grafted plant was regarded as one replicate) of all grafting combinations from each treatment group (control and 6 hours of chilling) were analyzed. Values are log_2_-median-transformed averages. **a** PC1 and PC2 scores for the averaged values of 542 metabolites, with four replicates per tissue for all grafts. **b** Top 10 metabolites from the positive and negative loadings of PC1 and PC2, respectively. PCA and heat maps of the top 10 metabolites from PC1 and PC2 were produced from ion intensity data using the online OmicShare Heatmap tools (http://www.omicshare.com/tools).

After filtering discrete samples, we performed PCA on a subset of the data including 30 samples from the first leaves of scions and 27 samples from roots of rootstocks after chilling stress and in the normal condition, which showed a clear separation between different tissues and species (Fig. 2a; Supplementary Data Fig. S2). Source (the first leaves of scions) and sink (roots of rootstocks) organs were separated by PC1, which explained 14.4% of the total variance in the data set, and the different species in different grafted combinations were separated by PC2, explaining a further 11.6% of the variance (Supplementary Data Table S3). We excluded samples Normal_root^Cmo/Cmo^, Normal_root^Csa/Cmo^, Cold_root^Cmo/Cmo^, and Cold _root^Csa/Cmo^ from further analysis because the data sets for metabolites in these groups contained contaminants and missing values. Due to the strong organ and species specificity of metabolite composition, we performed our further data processing and analysis separately with respect to the different organs in different species. The top 10 positive and negative loadings of PC1, distinguishing leaf and root organs, and PC2, distinguishing species, are shown in Fig. 2b. Metabolites corresponding to positive and negative loadings of PC1 showed high abundance in leaves and low abundance in Csa roots. In contrast, both positive and negative loadings of PC2 corresponded to metabolites that were more abundant in Csa leaves and less abundant in Cmo leaves and roots.

Hierarchical cluster analysis of log_2_-median-transformed data on the fold change (6 hours of chilling/no chilling) revealed substantial differences in three organs of four graft combinations (Supplementary Data Fig. S3a and Table S4). The eight clusters, containing 542 metabolites, were informative with regard to differential chilling-induced response in heterografts. After chilling stress, metabolites in cluster I, mainly including terpenoids, amino acids, and lipids such as 9,10-EOH (negative compound), showed significant decreases in the root of Cmo/Csa versus Csa/Csa; sugars (raffinose, negative compound) and flavonoid in cluster II showed significant increases in the leaf of Cmo/Csa versus Cmo/Cmo, and compounds involved in amino acid biosynthesis and metabolism were drastically reduced in the leaf of Csa/Cmo versus Csa/Csa. Simultaneously, certain metabolites such as α-isopropylmalate and acetyl phosphate in cluster II were markedly lower in the root of Cmo/Cmo. Certain metabolites in cluster III, mainly including amino acids, terpenoids, organic acids, and hormones, such as *N*^6^-(δ2-isopentenyl)-adenine, were also drastically reduced in leaves of Csa/Cmo versus Csa/Csa, while the metabolites involved in amino acid metabolism, linolenic acid metabolism, and sugar metabolism were prominently increased in Cmo/Cmo leaf, such as 5-oxoproline and (−)-jasmonic acid in the pathway of fatty acid biosynthesis. Metabolites in cluster IV, including fatty acyls, carbohydrates, terpenes (such as 2-*C*-methyl-d-erythritol 4-phosphate) and amino acids, were reduced in the root of Cmo/Csa versus Csa/Csa, while the metabolites of carbohydrates (kaempferol 3-*O*-glucoside) and secondary metabolites (quinolinate) were significantly increased in Csa/Cmo leaf. In cluster V, in the leaf of Csa/Cmo versus Csa/Csa, flavonoids, vitamins and cofactors, pantothenate, CoA biosynthesis (pantetheine), and lipids were all drastically reduced. In cluster VI, amino acid (*N*-acetyl-l-glutamic acid), fatty acyls, and alkaloids were decreased after chilling stress in Cmo/Csa leaves. By contrast, compared with the roots of Csa/Csa, metabolites in cluster VII, including carbohydrates, vitamins, and cofactor amino acids, were notably increased in the root of Cmo/Csa. It is worth noting that d-glucose, involved in both carbohydrate metabolism and linolenic acid metabolism in Cmo/Csa leaf tissues, was significantly reduced. In cluster VIII, some metabolites of alkaloids, terpenoids, and lipids were significantly decreased in the leaves of Cmo/Csa versus Cmo/Cmo. Compared with other grafted combinations, phenylpropane was specifically higher in the roots of Csa/Csa versus Cmo/Csa, but the amino acids of hydroxyproline and fatty acyls were specifically reduced in the leaf of Csa/Csa versus Csa/Cmo (Supplementary Data Fig. S3b). In summary, the metabolites with significant changes in homograft combinations were mainly distributed in clusters I, II, and III, while those in heterograft combinations were mainly distributed in clusters IV–VIII (Supplementary Data Fig. S3b). In summary, the metabolite profiling analysis showed that metabolic changes were specific to organs and species under early chilling stress.

### Transcript profiling reveals common and specific early chilling responses in different tissues of different graft combinations

**Figure 3:**
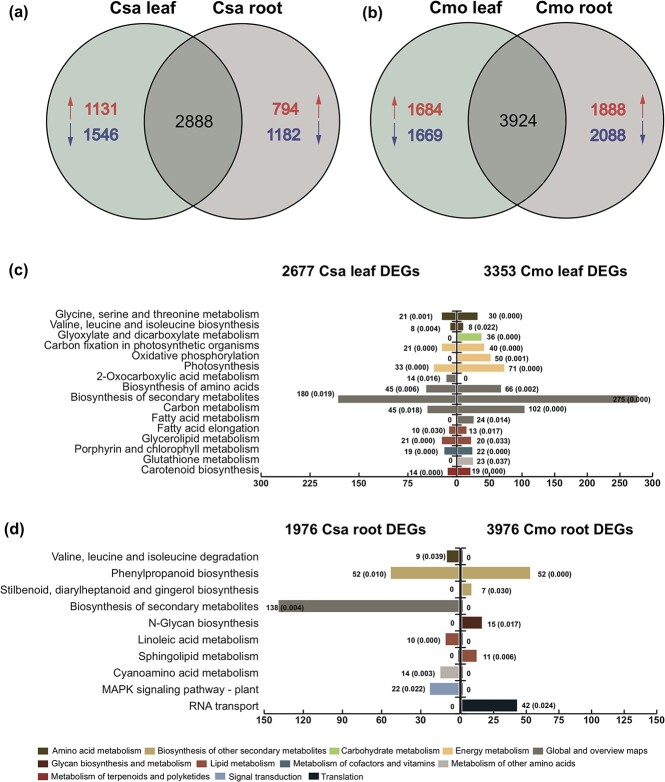
**a**, **b** Venn diagrams of transcripts that were significantly differentially expressed between heterografts and homografts under 6 hours of chilling conditions. Comparison of upregulated (red arrows) and downregulated (blue arrows) Csa DEGs (**a**) and Cmo DEGs (**b**) in leaf and root of heterografts and homografts. DEGs in (a) and (b) have value ≥2. **c**, **d** KEGG pathway analysis of Csa DEGs and Cmo DEGs in leaves (**c**) and roots (**d**). Different colors indicated different KEGG B classes. Three replicates of the pooled first true leaves and 9 or 10 individual seedlings per replicate were used for RNA-seq analysis under control condition and chilling stress for 6 hours.

First, transcript profiling performed in the same heterografted samples identified 2436 upregulated and 3129 downregulated Csa transcripts in the leaves, and 1910 upregulated and 2954 downregulated Csa transcripts in the roots, under early chilling stress. Meanwhile, we identified 4028 upregulated and 3249 downregulated Cmo DEGs in the leaves and 4152 upregulated and 3738 downregulated Cmo DEGs in the roots (Supplementary Data Fig. S4, Supplementary Data Table S5). Two thousand, eight hundred and eighty-eight Csa DEGs sharing an overlap between leaf and root were found to participate in the pathways for the biosynthesis of amino acids, biosynthesis of unsaturated fatty acids, and carbon metabolism (Fig. 3a; Supplementary Data Table S6), whilst the 3924 Cmo DEGs that overlapped between roots and leaves were enriched in pathways of α-linolenic acid metabolism, citrate cycle (TCA cycle), carbon fixation in photosynthetic organisms, and biosynthesis of amino acids (Fig. 3b; Supplementary Data Table S7).

Among the DEGs, 2677 Csa DEGs exclusively in leaves were enriched in the pathways of amino acid metabolism, energy metabolism, lipid metabolism, metabolism of terpenoids and polyketides, and cofactor and vitamin metabolism. Three thousand three hundred and fifty-three Cmo DEGs exclusively in leaf were enriched in the pathways of glyoxylate and dicarboxylate metabolism, oxidative phosphorylation, fatty acid metabolism, and glutathione metabolism. Fourteen Csa DEGs were found to specifically participate in 2-oxocarboxylic acid metabolism of the KEGG pathway (Fig. 3c; Supplementary Data Tables S6 and S7). Moreover, 1976 Csa DEGs exclusively expressed in roots were enriched in the pathways of valine, leucine, and isoleucine degradation, biosynthesis of secondary metabolites, linoleic acid metabolism, cyanoamino acid metabolism, and plant MAPK signaling, while 3976 Cmo DEGs exclusively in roots were enriched the pathways of stilbenoid, diarylheptane, and gingerol biosynthesis, *N*-glycan biosynthesis, sphingolipid metabolism, and RNA transfer (Fig. 3d; Supplementary Data Tables S6 and S7). These results indicated that the biological functional enrichment of Csa and Cmo DEGs were species- and tissue-specific under chilling stress.

### Many transcripts are directionally mobile in grafted cucumber and pumpkin seedlings in response to early chilling

**Figure 4:**
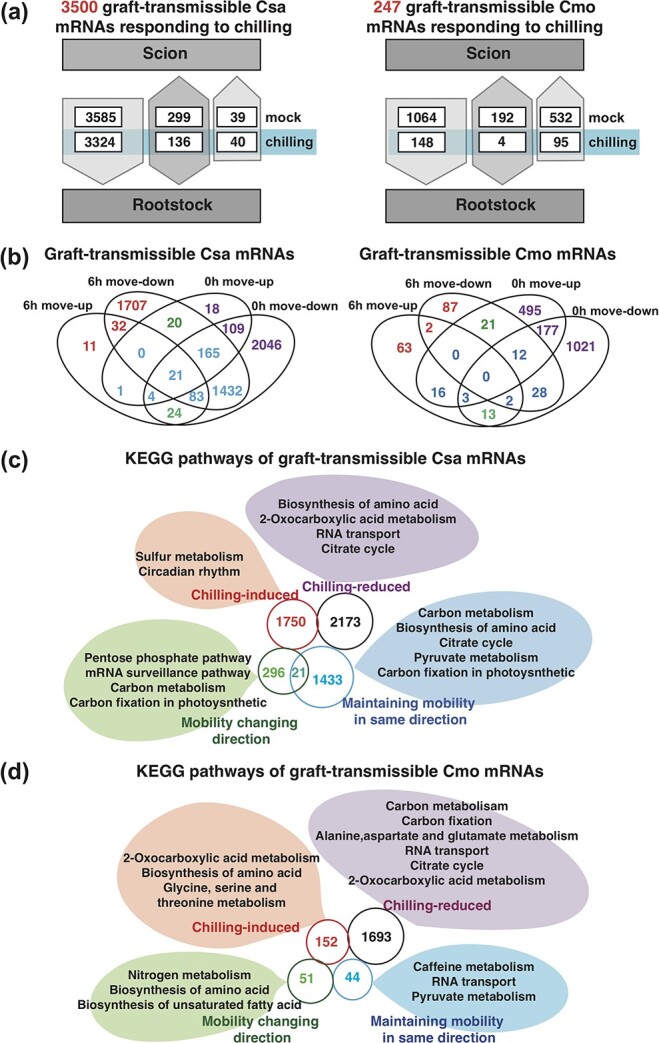
Analysis of mobile mRNAs between rootstock and scion in control and 6 hours of chilling conditions. **a** Numbers and movement directions of mobile mRNAs exchanged between shoot and root tissues in Csa and Cmo under chilling stress compared with control. **b** Venn diagrams of graft-transmissible Csa and Cmo mRNAs. **c**, **d** KEGG pathway analysis of Csa and Cmo mobile mRNAs. The mRNAs were classified into four types based on their direction of movement: chilling-induced (orange), chilling-reduced (purple), mobility changing direction (green), and mobility maintained in the same direction (blue).

To better verify the transportability of RNA signaling molecules in Csa/Cmo heterografts and reverse-grafting Cmo/Csa heterograft combinations, we devised a strategy based on hypocotyl grafting of size-matched Csa and Cmo seedlings with one true leaf. To analyze graft-transmissible Csa and Cmo transcripts present in the recipient heterografted tissues, we used the mobile transcript-calling strategy previously reported [48]. The mobile transcripts identified in rootstock and scion samples from reciprocally grafted seedlings allowed us to trace the direction of movement into heterologous tissues and the species dependency of this chilling-induced movement. First, we classified the directionality of movement of the identified mobile mRNAs into three categories by comparing Cmo mRNAs found in Csa scions grafted onto Cmo rootstock with those found in the reverse grafting combination after 6 hours of chilling or in the absence of chilling. We unambiguously assigned 3923 and 3500 Csa and 1788 and 247 Cmo transcripts in the no-chilling and 6 hours of chilling conditions, respectively. Among the 3500 early-chilling-induced mobile Csa mRNAs, 95.0% (*n* = 3324) showed unidirectional migration from Csa scion to Cmo rootstock, 1.1% (*n* = 40) migrated uniquely from Cmo rootstock to Csa scion, and 3.9% (*n* = 136) showed bidirectional movement. Likewise, among the 247 early-chilling-induced mobile Cmo mRNAs, 60.0% (*n* = 148) migrated from Cmo scion to Csa rootstock, 38.5% (*n* = 95) migrated from Cmo rootstock to Csa scion, and 1.6% (*n* = 4) underwent bidirectional movement. Comparing these numbers with those for mobile mRNAs from plants in the no-chilling condition, we found that, except for the bidirectional movement of Cmo mRNAs, all mobility of both Csa and Cmo mRNAs was induced by the chilling treatment (Fig. 4; Supplementary Data Table S8).

Furthermore, we classified the identified Csa and Cmo mobile mRNAs in different graft combinations with and without 6 hours of chilling in Fig. 4b into four categories: those undergoing chilling-induced mobility, chilling-reduced mobility, mobility changing direction, and mobility maintaining direction (Supplementary Data Table S9). KEGG enrichment analysis of these categories of mobile mRNAs was performed to determine their function classifications. The 1750 Csa and 152 Cmo mRNAs with chilling-induced mobility were most highly enriched in the sulfur metabolism and 2-oxocarboxylic acid metabolism pathways, respectively, while 1693 Cmo and 2173 Csa chilling-reduced mRNAs were highly enriched in the carbon metabolism and amino acid biosynthesis pathways. Interestingly, some mRNAs changed movement direction after chilling compared with the normal condition, including 51 Cmo mRNAs enriched in amino acid and unsaturated fatty acid biosynthesis, and 317 Csa mRNAs enriched in carbon metabolism. Moreover, 1433 Csa mRNAs and 44 Cmo mRNAs maintaining mobility in the same direction were enriched in pyruvate metabolism and carbon metabolism, respectively (Fig. 4c and d; Supplementary Data Table S10).

### Integrated analysis of transcript and metabolite profiles reveals that mobile mRNAs of pumpkin provided a link to metabolic changes in cucumber tissues of heterografts under the early chilling response

To determine whether there is a correlation among mobile mRNAs, transcriptional and metabolomic differences of heterografts and homografts at 6 hours of chilling stress, an analysis strategy was developed: first, three pairwise comparisons between them were performed based on KEGG pathways, then integration analysis of overlapped pathways was performed among the three pair comparisons.

Firstly, we investigated the correlation between mobile mRNAs and DEGs in the leaves and roots of heterografts and homografts with and without 6 hours of chilling stress. Of 152 chilling-induced mobile mRNAs from Cmo, 107 were related to Cmo DEGs (Supplementary Data Fig. S5a). Meanwhile, 152 chilling-induced Cmo mobile mRNAs were matched to 148 homologous Csa protein-encoding mobile mRNAs by orthology analysis with best hits. After filtering out duplications, 148 Csa mRNAs homologous to Cmo mobile transcripts were compared with Csa DEGs and were found to share 95 chilling-induced Cmo mobile mRNAs relating to Csa DEG changes (Supplementary Data Fig. S5b). A Venn analysis of 107 chilling-induced Cmo mobile mRNAs related to Cmo DEGs and 95 chilling-induced Cmo mobile mRNAs related to Csa DEGs indicated that 49 chilling-induced Cmo mobile mRNAs were related to both Csa and Cmo DEGs (Supplementary Data Fig. S5d). This set of 49 chilling-induced Cmo mobile mRNAs was enriched in the KEGG pathways of the metabolism of amino acids (glycine, serine, and threonine) and fatty acids (Supplementary Data Fig. S5g). Moreover, 481 chilling-induced Csa mobile mRNAs shared an overlap with 877 chilling-induced Cmo mRNA mobility-related Cmo DEGs and 772 chilling-induced mobile Csa mRNAs with Cmo DEGs change (Supplementary Data Fig. S5c, e, f). These 481 chilling-induced candidates were mainly enriched in the KEGG pathways of photosynthesis, sulfur metabolism, α-linolenic acid metabolism and so on (Supplementary Data Fig. S5h, Supplementary Data Table S11). In the same way, we then verified the characterization of the categories of chilling-reduced and mobility-direction-changed mRNAs. The Venn analysis result indicated that 949 chilling-reduced mobility of Csa mRNAs associated with Csa and CmoDEGs were enriched in arginine biosynthesis, carbon fixation, and phenylalanine, tyrosine, and tryptophan biosynthesis, while 628 chilling-reduced mobility of Cmo mRNAs associated with Csa and CmoDEGs were enriched in pathways of the TCA cycle, carbon fixation, and cysteine and methionine metabolism (Supplementary Data Fig. S6a–h, Supplementary Data Table S11). Moreover, 167 mobility-direction-changed Csa mRNAs associated with Csa and Cmo DEGs were enriched in the TCA cycle, carbon metabolism, and biosynthesis of amino acids, while 22 mobility-direction-changed Cmo mRNAs associated with Csa and Cmo DEGs were significantly enriched in unsaturated fatty acid biosynthesis (Supplementary Data Fig. S7a–h; Supplementary Data Table S11).

Secondly, we performed correlation analysis of DIMs and DEGs in the leaves and roots of heterografts and homografts due to chilling stress. Three hundred and seventy-eight metabolites were significantly correlated with 3925 Csa DEGs, and 324 metabolites were significantly correlated with 4039 Cmo DEGs (|cor| ≥0.95) (Supplementary Data Table S12). These DEGs significantly related to DIMs were mainly involved in the KEGG pathways of cofactor and vitamin metabolism, carbohydrate metabolism, terpenoid and polyketide metabolism, amino acid metabolism, and lipid metabolism (Supplementary Data Tables S13 and S14).

**Figure 5:**
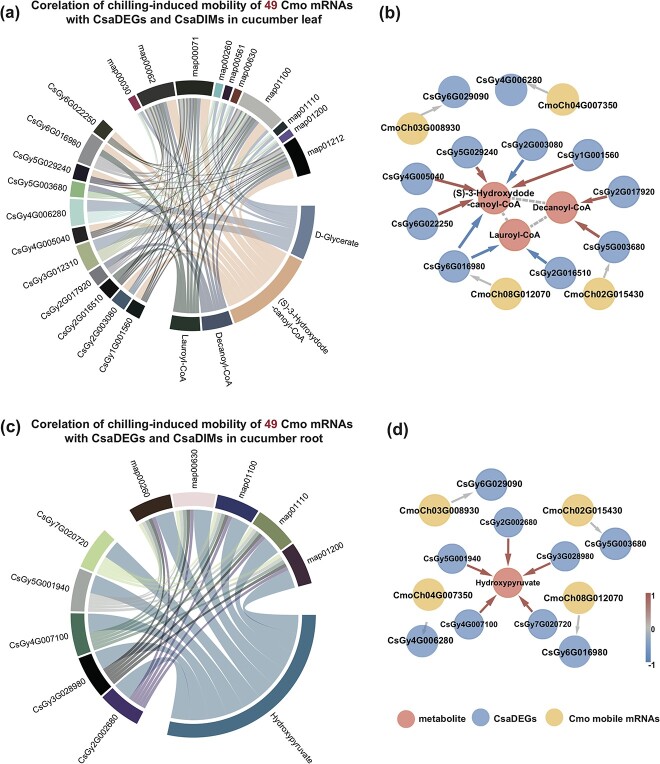
Integrated analysis of chilling-induced mobile mRNAs, DEGs, and DIMs. **a**, **c** Chord diagrams of chilling-induced mobile Cmo mRNAs, metabolites, Csa DEGs, and metabolic pathways in Csa leaves (**a**) and roots (**c**) of heterografts, created with the circlize R package. **b**, **d** Networks (**b**, leaves; **d**, roots) involving the metabolites (*S*)-3-hydroxydodecanoyl-CoA, decanoyl-CoA, lauroyl-CoA, and hydroxypyruvate and related Csa DEGs and chilling-induced homologous mobile Cmo mRNAs. The Csa DEGs in Csa leaves of heterografts associated with at least one chilling-induced mobile Cmo mRNAs are visualized with Cytoscape (version 3.8.1). Red circles indicate the two metabolites, blue circles indicate Csa DEGs, and orange circles indicate Csa DEG-coordinated homologous Cmo mobile mRNAs. Arrows (red for positive and blue for negative correlation, gray for the corresponding relationship) indicate the correlation between metabolites and Csa DEGs.

Then, to investigate the effect of specific chilling-induced mobile Cmo mRNAs on the transcriptomic and metabolomic changes in Csa tissues in Csa/Cmo and Cmo/Csa, we first analyzed the relationship between mobile mRNA-related Csa DEGs and chilling-induced mobile Cmo mRNAs based on KEGG pathways. Four of 49 chilling-induced mobile Cmo mRNAs and 16 of 3925 Csa DEGs significantly correlated with Csa DIMs were found to be enriched in glycine, serine, and threonine metabolism (ko00260) and fatty acid degradation metabolism (ko00071). Therefore, in the following studies, we found that 5 DIMs (d-glycerate, (*S*)-3-hydroxydodecanoyl-CoA, decanoyl-CoA, lauroyl-CoA, and hydroxypyruvate) and 16 Csa DEGs with significant changes were enriched in the focused pathways of glycine, serine, and threonine metabolism and fatty acid metabolism in Csa leaves and roots in response to chilling stress. The integrated analysis of the five key metabolites, 16 Csa DEGs, and 4 mobile mRNAs using Cytoscape based on the KEGG pathway in Csa tissues in response to chilling revealed a significant correlation (*P* < .05) between the three in glycine, serine, and threonine metabolism (ko00260) and fatty acid degradation (ko00071) (Supplementary Data Table S15). In Csa leaves of Csa/Cmo in response to chilling, a significant correlation between 4 DIMs (d-glycerate, decanoyl-CoA, lauroyl-CoA, and (*S*)-3-hydroxydode-canoyl-CoA) and 11 Csa DEGs were enriched in the pathways of the pentose phosphate pathway (ko00030), glycine, serine and threonine metabolism (ko00260), fatty acid degradation (ko00071), glycerolipid metabolism (ko00561), glyoxylate and dicarboxylate metabolism (ko00630), metabolic pathways (ko01100), biosynthesis of secondary metabolites (ko01110), fatty acid metabolism (ko01212), fatty acid elongation (ko00062), and carbon metabolism (ko01200) (Fig. 5a). In the pathway of fatty acid degradation, 4 mobile Cmo mRNAs (CmoCh03G008930, CmoCh04G007350, CmoCh08G012070, and CmoCh02G015430), 11 Csa DEGs, and 3 DIMs (decanoyl-CoA, (*S*)-3-hydroxydode-canoyl-CoA, and lauroyl-CoA) were highly correlated (Fig. 5b). In Csa roots of Csa/Cmo in response to chilling, the metabolite of hydroxypyruvate and five CsaDEGs were highly correlated into five main metabolic pathways of glycine, serine, and threonine metabolism (ko00260), biosynthesis of secondary metabolites (ko01110), and carbon metabolism (ko01200) (Fig. 5c). In the pathway of glycine, serine, and threonine metabolism, these four chilling-induced mobile Cmo mRNAs, nine Csa DEGs, and DIM of hydroxypyruvate were highly correlated (Fig. 5d; Supplementary Data Table S16). Additionally, in Cmo scion of Cmo/Csa, 4 of 481 chilling-induced mobility of Csa mRNAs, 4 DIMs (*N*-acetyl-l-glutamate-5-semialdehyde, succinate, methyl jasmonate, and (9*Z*,15*Z*)-(13*S*)-12,13-epoxyoctadeca-9,11,15-trienoic acid) were highly correlated into two main metabolic pathways of sulfur metabolism (ko00920) and arginine and proline metabolism (ko00330) (Supplementary Data Fig. S9, Supplementary Data Table S11).

To investigate the effects of chilling-reduced mobility and mobility-direction-changed mRNAs on transcriptomic and metabolomic changes of heterografts, by using the same strategy we found that 7 of 628 chilling-reduced mobility of Cmo mRNAs, 4 DIMs (oxaloacetate, acetaldehyde, *S*-adenosyl-methioninamine, and 2-oxogulatrate) were associated with DEGs enriched in the pathways of glyoxylate and dicarboxylate metabolism (ko00630), cysteine and methionine metabolism (ko00270), pyruvate metabolism (ko00620), and the TCA cycle (ko00020) in Csa scion (Supplementary Data Fig. S8a and b). In Csa rootstock, only one chilling-reduced mobility of Cmo mRNA (CmoCh08G006150) and one DIM of 2-oxogulatrate associated with DEGs were enriched in the glyoxylate and dicarboxylate metabolism pathway (ko00630) (Supplementary Data Fig. S8a and b). Meanwhile, in Cmo scion of Cmo/Csa, 10 of 949 chilling mobility of Csa mRNAs, 5 DIMs (succinate, phenylacetaldehyde, (*S*)-malate, and 4-coumarate, phenylpyruvate) were associated with DEGs mainly enriched in the pathways of phenylalanine, tyrosine, and tryptophan biosynthesis (ko00400), carbon fixation in photosynthetic organisms (ko00710), and porphyrin and chlorophyll metabolism (ko00860) (Supplementary Data Fig. S8c and d). Furthermore, in Cmo scion of Cmo/Csa, 4 of 167 mobility-direction changed Csa mRNAs and 8 DIMs (citrate, isocitrate, 2-oxoglutarate, (*R*)-2-methylmalate, (*S*)-malate, phenylpyruvate, shikimate, and (2S)-2-isopropyl-3-oxosuccinate) were associated with DEGs enriched in the pathways of glyoxylate and dicarboxylate metabolism (ko00630) and alanine, aspartate, and glutamate metabolism (ko00250) (Supplementary Data Fig. S9a and b). However, we did not identify any candidates in 22 mobility-direction-changed Cmo mRNAs that were correlated with DEGs and DIMs in Csa tissues.

In summary, the integration of Csa DIMs, Csa DEGs, and mobile Cmo mRNA profiles indicated that mobility of certain Cmo mRNAs strongly associated with transcriptional and metabolic changes in the response to chilling stress in Csa tissues of Csa/Cmo and Cmo/Csa grafts compared with Csa/Csa. To find out how mobile Cmo mRNAs acted in the metabolic response of Csa in chilling stress, we focused on the chilling-induced mobility of Cmo mRNAs associated with four metabolites (hydroxypyruvate, decanoyl-CoA, lauroyl-CoA, and (*S*)-3-hydroxydode-canoyl-CoA) in glycine, serine, and threonine metabolism and fatty acid degradation in both sensitive Csa leaves and roots of Csa/Cmo and Cmo/Csa under the chilling condition in a further study.

### Pumpkin mobile mRNAs are involved in the metabolic pathways of glycine, serine, and threonine synthesis and fatty acid β-oxidative elongation in grafted cucumber under the chilling response

**Figure 6:**
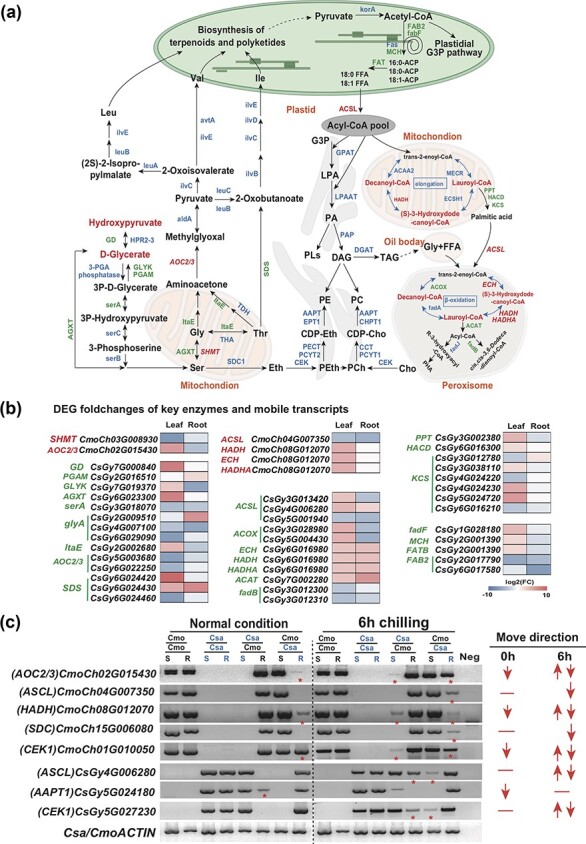
Schematic metabolic pathway of chilling-induced Cmo mobile mRNAs participating in amino acid synthesis and fatty acid metabolism in grafted cucumber. **a** Hydroxypyruvate is a product of the pentose phosphate cycle pathway, which is catalyzed by glycerate dehydrogenase (GD, hprA, EC 1.1.1.29) and glyoxylate/hydroxypyruvate reductase (HPR2-3, EC 1.1.1.81) to generate d-glycerate. d-Glycerate is further catalyzed by d-glycerate 3-kinase (GLYK, EC 2.7.1.31) and 2,3-bisphosphoglycerate-dependent phosphoglycerate mutase (PGAM, EC 5.4.2.11) to generate 3-phospho-d-glycerate, which is a precursor for the synthesis of serine. Serine is converted to glycine by glycine hydroxymethyltransferase (glyA, SHMT, EC 2.1.2.1, CmoCh03G008930). Glycine is converted to threonine under the catalysis of threonine aldolase (TA, ItaE, EC 4.1.2.48) and l-threonine aldolase (THA, EC 4.1.2.5). Glycine undergoes a series of catalytic reactions to generate aminoacetone, which was catalyzed by primary amine oxidase (AOC3, AOC2, tynA, EC 1.4.3.21, CmoCh02G015430) to produce methylglyoxal (pyruvaldehyde), and it could form pyruvate after further reactions to generate 2-oxoisovalerate and 2-oxobutanoate, which can also be generated by l-threonine ammonia-lyase (SDS, EC 4.3.1.19) catalyzed by threonine. These metabolites can further synthesize branched-chain amino acids (leucine, valine, and isoleucine) and a variety of secondary metabolites, including glycosides, glucose isothiocyanates, acyl sugars, and some volatiles. In the synthesis process of fatty acids, long-chain acyl-CoA synthetase (ACSL, fadD, EC 6.2.1.3, CmoCh04G007350) catalyzes the production of acyl-CoA (acyl-CoA), and further catalyzes DAG, which is a common substrate for different types of glycerolipids, including galactolipids, sulfur, phospholipids, and triacylglycerols. In mitochondria, decanoyl-CoA, lauroyl-CoA and (*S*)-3-hydroxydodecanoyl-CoA are metabolized by acetyl-CoA acyltransferase 2 (AACA2, EC 2.3.1.16), 3-hydroxyacyl-CoA dehydrogenase (HADH, EC 1.1.1.35, CmoCh08G012070), mitochondrial enoyl-[acyl-carrier protein] reductase (MECR, NRBF1, EC 1.3.1.- 1.3.1.38), and enoyl-CoA hydratase (ECSH1) to promote the elongation of fatty acids (4 < *n* < 16 in mitochondria, *n* > 16 in the endoplasmic reticulum). Then, under the catalysis of palmitoyl-protein thioesterase (PPT, EC 3.1.2.22), very-long-chain (3R)-3-hydroxyacyl-CoA dehydratase (HACD, PHS1, PAS2, EC 4.2.1.134), and 3-ketoacyl-CoA synthase (KCS, EC 2.3.1.199), the fatty acid signal is transmitted by mitochondria to peroxisomes participating in the metabolic process of fatty acid β-oxidation degradation. The red-colored genes represent chilling-induced mobile Cmo mRNAs, the green-colored genes represent Csa DEGs with significant correlation with Csa DIMs (*P* < .05), and the blue-colored enzymes represent key enzymes in these metabolic networks. The red-colored metabolites represent Csa DIMs with significant changes in the Csa of Csa/Cmo and Cmo/Csa compared with the same tissue of Csa/Csa. **b** Heat map of log_2_-median-transformed average fold change in either Csa or Cmo leaves and roots of heterografts compared with the same tissues in homografts under control and 6 hours of chilling stress conditions. **c** RT–PCR verification of predicted mobile mRNAs. The red asterisks indicate mobile mRNA direction after chilling stress. Red arrows indicate mobile direction and red lines indicate unidentified mobility. Each sample included 9 or 10 biological replicates and three to five technique replicates.

The metabolite measurements provided evidence that mobile mRNAs of Cmo associated with metabolic changes in the pathways of glycine, serine, and threonine metabolism and fatty acid degradation in Csa leaves and roots of Csa/Cmo and Cmo/Csa under chilling stress (Fig. 6a); however, to further study the potential function of mobile mRNAs, we were still interested in finding out what exact reactions, the mobile Cmo mRNAs are involved in and how they regulate the metabolic changes.

In the Csa leaf of Csa/Cmo, mobile Cmo mRNA of *ACSL* (CmoCh04G007350), the enzyme associated with *CsaDED* of *Fab*, catalyzed the formation of acyl-CoA to provide precursors for lipid synthesis in the endoplasmic reticulum. Furthermore, mobile Cmo mRNAs of *HADH* (CmoCh08G012070) and *ECH* (CmoCh08G012070), associated with Csa DEGs of *PPT*, *HACD*, *KCS*, *ACOX*, and *ACAT*, related to the Csa DIMs of decanoyl-CoA, lauroyl-CoA, and (*S*)-3-hydroxydodecanoyl-CoA, were found to be involved in both the metabolic reactions of fatty acid (4 < *n* < 16) elongation in mitochondria and fatty acid β-oxidation degradation in peroxisomes. Simultaneously, in the Csa leaf of Cmo/Csa, mobile Cmo mRNAs of *AOC3* (CmoCh02G015430) and *SHMT* (CmoCh03G008930) associating Csa DEGs of *TA*, *AGXT*, *SDS*, *GD*, *GLYK*, *PGAM*, and *serA* participated in the biosynthesis of D-glycerate and hydroxypyruvate, and then providing precursors for serine, glycine, and pyruvate synthesis (Fig. 6b; Supplementary Data Table S16).

Based on our results, we performed RT–PCR verification on the predicted mobile mRNA by using specific primers as described previously [48]. RT–PCR indicated that chilling triggered the upward mobility of *CmoAOC2*/*3* and *CmoHADH* from Cmo root to Csa shoot, the downward mobility of *CmoACSL* and *CmoSDC* from Cmo shoot to Csa root, and the bidirectional mobility of *CsaACSL* mRNA. However, chilling also reduced the downward mobility of *CsaAAPT1* from Csa shoot to Cmo root, confirming that chilling can also affect the mobile capacity and movement direction of mRNA (Figs 4c and d and 6c; Supplementary Data Fig. S10). The result suggested that the mobile mRNAs of key enzymes and key metabolites involved in the transitional pathway, glycerophospholipid metabolism (ko00564), of glycine, serine, and threonine metabolism, and fatty acid degradation were highly related to Csa chilling tolerance.

**Figure 7:**
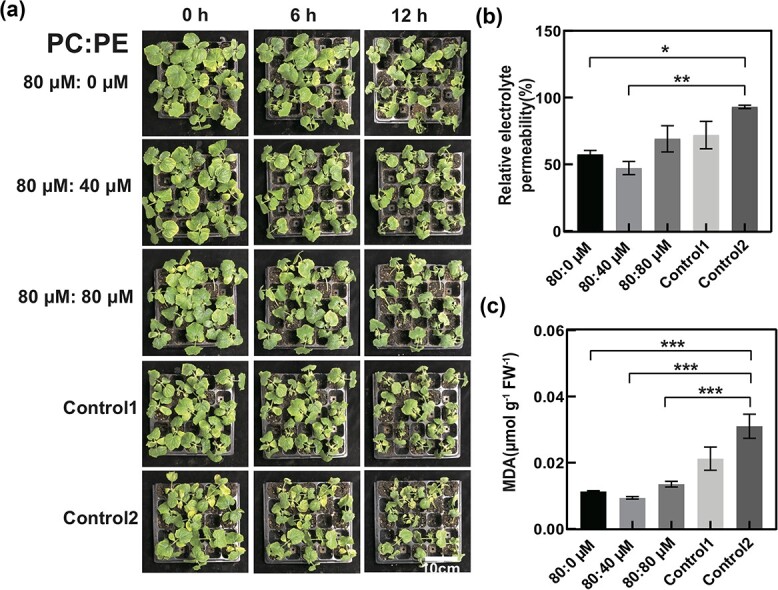
Effect of exogenous metabolite application on the chilling tolerance of Csa seedlings. **a** Exogenous application of combination of phosphatidylcholine (PCho) and phosphatidylethanolamine (PEth) in two-leaf-stage Csa seedlings. PCho and PEth were dissolved in 0.1% ethanol and 0.1% chloroform. Therefore, control 1 was the 2:1 (v:v) mixture solution of 0.1% ethanol and 0.1% chloroform with 0 μM PCho and 0 μM PEth. Control 2 was the solution of 0.1% chloroform with 0 μM PEth. (b) REP and (c) MDA content of first leaves under different conditions after chilling treatment for 12 hours. Six individual plants at each time point were used for analysis. Student’s *t*-test was used to analyze significant differences. Asterisks indicate a significant difference at each time point compared with normal (^***^P < .001, ^**^P < .05, ^*^P < .01).

Then, to verify whether the key metabolites in these pathways have potential to regulate Csa chilling tolerance, we exogenously supplied different concentrations of key metabolites of phosphatidylcholine (PCho), phosphatidylethanolamine (PEth), choline (Cho), and ethanolamine (Eth), which were all products of glycine, serine, and fatty acid metabolism in two-leaf-old Csa seedlings, for 4 days before chilling treatment. After chilling treatment, the phenotypic changes and relative electrolyte permeability (REP) results suggested that the effects of different concentrations of the four metabolites on the chilling tolerance of Csa differ. The application of Cho and PEth performed negative regulation on 24-hour chilling tolerance in Csa. REP was significantly higher in Csa under 80 μM PEth solution compared with chloroform control, and also REP was significantly higher in Csa treated with 20 μM Cho compared with other concentrations. Meanwhile, the application of Eth and PCho in Csa under 24 hours of chilling exhibited nearly the same change trend with concentration, i.e. REP was significantly lower in the 60 μM Eth treatment (Supplementary Data Fig. S11). The result allowed us to focus on the positive effect of 80 μM PEth and the negative effect of PCho in the pathway of glycerophospholipid metabolism (ko00564) on Csa chilling responses in a further study. Thus, we performed an examination with different concentration ratios of PEth and PCho combinations in Csa seedlings chilling responses. Analysis of REP and malondialdehyde (MDA) content indicated that the PCho:PEth ratio of 80 μM:40 μM was the best combination for significantly improving Csa chilling tolerance (Fig. 7).

**Figure 8:**
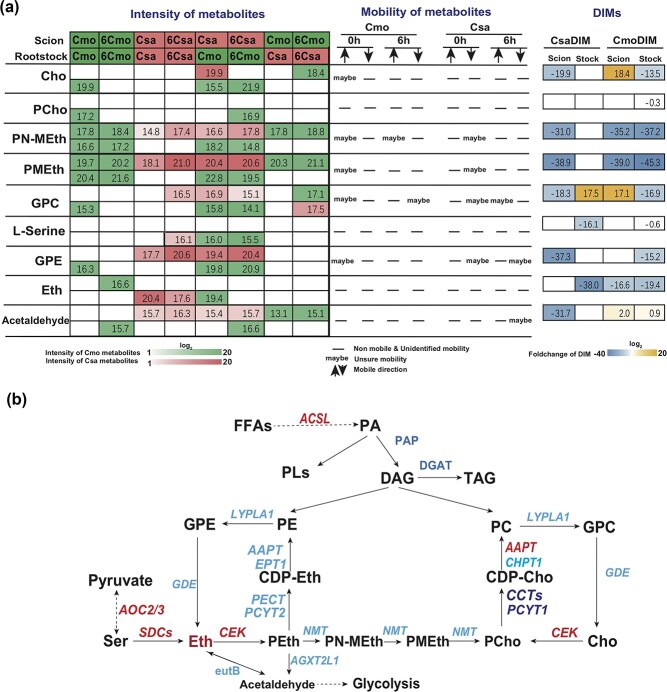
Potential mobility of metabolites, DIM, and key mobile mRNAs in the pathway of glycerophospholipid metabolism in grafted Csa responding to chilling. **a** Potential mobility of metabolites and DIMs and detected intensity of metabolites in the pathway of glycerophospholipid metabolism. **b** Scheme showing the links between metabolite accumulation and mRNA mobility in glycerophospholipid metabolism. Red letters indicate mobility of mRNA triggered by chilling, dark blue letters indicate reduced mobility by chilling, and light blue letters indicate the genes of enzymes. PN-MEth, *N*-methylethanolamine phosphate; PMEth, phosphodimethylethanolamine; GPC, sn-glycero-3-phosphocholine; GPE, sn-glycero-3-phosphoethanolamine; LYPLA1, lysophospholipase I (EC 3.1.1.5); GDE, glycerophosphodiester phosphodiesterase (EC 3.1.4.46); NMT, phosphoethanolamine *N*-methyltransferase (EC 2.1.1.103); AGXT2L1, ethanolamine-phosphate phospholyase (EC 4.2.3.2); eutB, ethanolamine ammonia-lyase large subunit (EC 4.3.1.7).

To study the mobility of mRNAs involved in the chilling response of grafted Csa, excluding the impact of metabolite transportation, we looked in detail into the Total Ion Current (TIC) changes of metabolites involved in the pathway of glycerophospholipid metabolism (ko00564). Of all the metabolites that could be detected in the shoot and root of all graft combinations, it was found that the metabolites of l-serine, PCho and Eth were identified as not mobile in either Csa or Cmo (Fig. 8a). The movement of some metabolites maybe triggered in Csa under chilling stress, such as GPC, sn-glycero-3-phosphocholine (GPC), sn-glycero-3-phosphoethanolamine (GPE), and acetaldehyde, while movement of some metabolites maybe reduced in Cmo due to chilling, such as N-Methyle thanolamine phosphate (PN-Meth), Cho, phosphodimethylethanolamine (PMEth), and sn-glycero-3-phosphoethanolamine (GPE) (Fig. 8a). By integration analysis of metabolite transport, mRNA mobility, and DIMs we could confirm the potential role of mobile mRNAs in regulating the intensity of metabolites changes in responding to chilling. For example, based on the exclusion of non-mobile Eth in chilling, we found that the intensity of Eth decreased in both Cmo root of Csa/Cmo and in Csa root of Csa/Csa, but increased in Cmo shoot of Cmo/Cmo under chilling (Fig. 8a), while exogenous application indicated that Eth improved Csa tolerance (Fig. 7b). The integrated result allowed us to speculate that some downstream mRNAs moving to Cmo root of Csa/Cmo may contribute to Eth degradation or metabolism *in vivo*. RT–PCR of *CEK1* movement confirmed our speculation that *CsaCEK1* moved downward from Csa shoot to Cmo root under the chilling condition. Furthermore, PCho was identified as non-mobile in all conditions, and the intensity of PCho in Cmo root of Cmo/Cmo was decreased, indicating that PCho is sensitive to chilling (Fig. 8a). The intensity of PCho was highly induced in Cmo root of Csa/Cmo, so we speculated that some upstream mRNA candidates may move from Csa shoot into Cmo root and then increase the biosynthesis of PCho, or downstream mRNAs with decreased mobility capacity may move from Csa shoot into Cmo root, and then increase the accumulation of PCho (Fig. 8b). RT–PCR verification of increased mobility of *CsaCEK1* and decreased mobility of *CsaCCT1* and *CsaPCYT1* under chilling proved our speculation that *CsaCEK1*, *CsaCCT1*, and *CsaPCYT1* play an important role in PCho accumulation and Csa chilling response regulation (Fig. 6c; Supplementary Data Fig. S10).

From the above results we concluded that the application of mobile mRNAs has potential to contribute to the complex metabolic networks of glycine, serine, and threonine biosynthesis and fatty acid β-oxidative degradation metabolism in Csa heterografts in the response to chilling.

## Discussion

### Grafting improves the ability of precursors of amino acid and lipid biosynthesis to supply energy for cucumber in early chilling stress

Grafting is generally used to produce various physiological effects on the growth and stress tolerance of horticultural crops, especially on chilling tolerance. It has been shown that chilling stress reduces the stability of the membrane ultrastructure of thylakoid and chloroplast, resulting in decreased photosynthetic efficiency [49]. In our study, we found that grafting Csa onto Cmo rootstock resulted in a higher transpiration rate and increased the accumulation of chlorophyll a, chlorophyll b, and carotenoid in Csa source leaf, resulting in improved chilling tolerance (Fig. 1; Supplementary Data Table S1). This may be explained by Cmo roots with strong vigor maintaining plant nutrient and water transport to combat dehydration,leading to a requirement for more carbohydrates in the source to restore the sink in Csa/Cmo compared with Csa/Csa. In Cmo/Csa under the chilling condition, compared with Cmo/Cmo the reverse situation holds. The enrichment of Csa DEGs in carbon fixation and photosynthesis also supported the idea that the effect of Cmo in improving Csa chilling tolerance involved photosynthesis improvement and maintenance of the source–sink balance (Fig. 3c), which is in line with previous studies [50–52].

When we looked in detail into the transcriptional and metabolic changes in Csa/Cmo and Cmo/Csa compared with Csa/Csa, we found that DEGs and DIMs in Csa tissues accumulated mainly in the pathway of fatty acid biosynthesis and metabolism in both leaves and roots (Fig. 3; Supplementary Data Tables S8 and S13). As is well known, when plants are subjected to external cold stress, the carbohydrate assimilation rate will be reduced, accompanied by reduced efficiency of the calvin cycle [53] and decreased fluidity of the plasma membrane, which is easily destroyed [54–56], resulting in an increase in membrane permeability, relative electrolyte leakage, and intracellular ion imbalance [57]. In this respect, our results indicated that lipid metabolism is the first player in the chilling response in Csa/Cmo and Cmo/Csa heterografts compared with Csa/Csa homografts [29, 58–62].

It is worth noting that our study specifically found that the integrated DEGs and DIMs were also enriched in the pathways of amino acid metabolism and glycine, serine, and threonine metabolism in both leaves and roots of Csa tissues when Csa/Cmo and Cmo/Csa were subjected to chilling stress (Fig. 5; Supplementary Data Table S14). It has been shown that abiotic stress directly inhibits amino acid biosynthesis and metabolism [63]. For example, proline synthesis is strongly induced during osmotic stress, leading to an increase in proline content [64]. Glycine and serine are precursors of chlorophyll, glutathione, tryptophan, phospholipids, and Eth. They affected the synthesis of PE and the composition of phospholipids and the plasma membrane [65]. In chilling stress conditions, amino acids can be used as alternative substrates for mitochondrial respiration [66–68]. Our results allowed us to conclude that grafting gives access to stored precursors for the biosynthesis of amino acids and lipids to supply energy and prepare for rapid recovery of plant metabolism under sudden chilling stress.

### Mobile mRNAs are involved in cold-responsive signaling exchanges between scion and rootstock in grafted cucurbits

The interactions between scions and rootstocks are complex. An increasing number of studies have been attempting to uncover the processes involved in the phenotypic and physiological changes induced by grafting [18, 43, 69–71]. These mechanisms influenced both root and shoot function, and the interconnectedness of the factors implicated (rootstock, scion, and environment) may provide singular contributions to phenotypic adaptation [21]. Currently, beyond its use in horticultural production, grafting has achieved considerable prominence as a research tool for its ability to isolate signaling mechanisms related to root–shoot communication and reveal large-scale, long-distance movement of mRNAs via the phloem [44, 46–48, 72–74]. However, little is known about mobile mRNA signals responding to chilling.

To make sure of the accuracy of mobile mRNA calling, firstly we screened mobile mRNAs based on next-generation sequence data filtered by 150-bp reads [44, 46, 48]. For example, if we would like to identify Csa mobility from Csa root to Cmo scion, we first have to compare reads in a sample of Cmo scion in Cmo/Csa with reads in Cmo/Cmo. That is, if reads come from the sample of Cmo scion in Cmo/Csa heterograft, they have to be BLASTed with reads of Cmo scion in Cmo/Cmo to screen out SNPs, but not full-length nucleotide sequences of genes. In fact, the BLAST database does not include any gene that is completely identical in Csa and Cmo. The Cmo has a large genome with 20 pairs of chromosomes, compared with Csa’s 7 pairs. There will have been a large number of synonymous mutations in the differentiation process of this species 3–20 million years ago, so it is difficult to find genes that are exactly the same in sequence between the two species. In our study, we completely established that systemic mobility of large-scale mRNAs as an early chilling response existed in grafted Csa and Cmo seedlings. The mobility of mRNAs between distant organs induced a cascade of signaling agents involved in metabolomic changes in the specific tissues to which the various graft-transmissible mRNAs are being delivered (Fig. 4). In previous studies, the existence of large-scale transport of mRNAs raised questions regarding their functionality; the mobility of mRNA molecules from companion cells into sieve tubes via plasmodesmata is a purposeful or aimless event is still in dispute. If transported mRNAs function through translation in their destination cells, their function and fate can then be understood by analyzing their protein products via proteomics or metabolomics [74]. GO and KEGG analyses indicated that such mobile mRNAs are involved in a broad range of biological processes and molecular functions [44, 74] but are over-represented in many basic cellular activities, as well as stress- and signaling-related responses, and in particular hormone metabolism and signaling [44, 46, 75]. Many systemic mobile mRNAs played specific physiological roles in such areas as photosynthesis/carbon metabolism and nutrient transport in recipient young leaves and roots [44, 48].

The characteristics of different types of mobile mRNAs in responding to chilling indicated that the migration of large-scale mobile mRNAs was not aimless; in particular, chilling-induced Cmo mRNAs were enriched in the pathway of glycine, serine and threonine metabolism (Fig. 4). The integration of Csa DIMs and Csa DEGs was also enriched in the same pathway, and some homologous genes between Cmo and Csa can be found to be identical (Fig. 5; Supplementary Data Table S15). The integrated analysis in our study linking mobile mRNAs, DIMs, and DEGs revealed that there indeed existed chilling-responsive signaling exchanges between tolerant Cmo with sensitive Csa, and systemic mRNAs were potentially involved in the biological processes of chilling alleviation. This conclusion provides more support for focusing on the potential function of selective mobile mRNAs in the regulation of plant growth and stress tolerance in the future.

### Mobile mRNAs in grafted cucumber responding to chilling stress were enriched in the metabolomic pathway of glycine, serine, and threonine metabolism and fatty acid β-oxidation degradation

In addition, the most important aspect of our study is that we addressed more relationships between mobile mRNAs that participated in the synthesis of glycine, serine, and threonine and fatty acid β-oxidative degradation (Figs 6a and b and 8; Supplementary Data Table S16). For example, in the reaction of glycine to serine, one of the key enzymes, serine hydroxymethyltransferase (glyA, SHMT, EC 2.1.2.1) catalyzed the mutual conversion of glycine and serine, and provided the activation required for the synthesis of nucleic acids and proteins and the one-carbon metabolism pathway [76]. SHMT participated in the photorespiration pathway of plant oxygen-containing photosynthetic organisms [77] and played a vital role in drought [78], high salinity [79], high Zn toxicity [76], and other abiotic stresses. AtSHMT1 was also involved in HCHO glycoassimilation [80]. Thus, we speculated that chilling-induced mobile *CmoSHMT* mRNA (CmoCh03G008930) may be involved in the mutual conversion of serine and glycine in grafted Csa, and promote photorespiration to enhance the tolerance of Csa to chilling stress.

Primary-amine oxidase (AOC3, AOC2, tynA, EC 1.4.3.21) is a heterogeneous enzyme, including copper amine oxidases (CuAOs) and flavin-containing polyamine oxidase (PAO), which catalyzes the deamination of polyamines (PAs) to amino aldehydes. It was found that copper amine oxidase (AtCuAO) in plants regulated H_2_O_2_ related to abscisic acid (ABA)-induced stomatal closure [81], and participated in wound healing, defense against pathogens [82], and indole-3-acetic acid (IAA)/jasmonic acid/ABA signal transduction pathways [81], such as the closure of stomata induced by ABA [83, 84]. In our results, we speculated that chilling-induced mobile Cmo mRNA of *CuAO* participated in the conversion of aminoacetone to pyruvaldehyde. By oxidizing polyamines to amino aldehydes, it may synthesize H_2_O_2_ and participate in the complex network of reactive oxygen species (ROS) signaling in response to chilling stress.

Long-chain acyl-CoA synthetases (ACSLs) are a group of rate-limiting enzymes in fatty acid metabolism that catalyze the synthesis of long-chain acetyl-CoAs. LACS2 (LONG-CHAIN ACYL-COA SYNTHETASE) in *Arabidopsis* is involved in unsaturated linolenic-CoA synthesis, which further increases the permeability of the stratum corneum of plant cells and thereby participates in the hypoxia signaling pathway mediated by ethylene response transcription factors [85]. Transcriptome analysis of waterlogged Csa showed a significant difference in the expression level of LACS6 in response to waterlogging stress between sensitive and tolerant Csa varieties [86]. This means that *LACS*s were responding to hypoxic stress caused by excessive water in the soil. Anoxia-induced plant cell ATP levels decreased to reduce the activity of long-chain ACSL, leading to change in the acyl-CoA pool [87]. Transcriptomic analysis at different developmental stages indicated that upregulation of LACS8 promoted the transportation of fatty acid precursors to the endoplasmic reticulum in wheat [88]. Our results allowed us to assume that chilling-induced mobile Cmo mRNA of *ACSL* (CmoCh04G007350) may participate in accumulation of the Csa acyl-CoA pool, thereby regulating subsequent fatty acid elongation metabolism. Furthermore, the metabolites of decanoyl-CoA, lauroyl-CoA, and (*S*)-3-hydroxydodecanoyl-CoA participated in the fatty acid elongation pathway under the catalysis of different enzymes, such as HADH (CmoCh08G012070). At the same time, the three metabolites were also involved in the fatty acid β-oxidation degradation pathway in peroxisomes; in this process enoyl-CoA hydratase (ECH, paaF, CmoCh08G012070) upregulated the accumulation of the unsaturated fatty acid arachidonic acid (ARA) [89]. HADHA (CmoCh08G012070) and HADH (CmoCh08G012070), the key enzymes in fatty acid β-oxidative degradation, were also involved in further jasmonic acid biosynthesis.

There may be more questions regarding the relations between metabolite transport and mRNA delivery (Fig. 8). The results presented here demonstrate that chilling induces the movement of mobile mRNAs linked to glycine, serine, and threonine synthesis and fatty acid β-oxidative degradation between rootstock and scion in heterografted Csa, increasing the accumulation of metabolites related to fatty acid β-oxidative degradation, and thereby improving chilling tolerance in these plants, e.g. increasing the accumulation of PCho and PEth (Fig 7; Supplementary Data Fig. S11). The significance of the study was to reduce the scope for searching for potential functional mobile mRNAs and helping to pinpoint the target physiological process. Prior to exploring target gene function by using genome-edited plants, the present results can help in speculation about the signaling function of mobile mRNAs that participate in chilling tolerance in Csa.

Thus, the processes involved in scion–rootstock interactions and the resulting graft-induced phenotypic and physiological changes can be explained by the functional mobility of protein-encoding mRNA transcripts.

## Materials and methods

### Plant materials, growth conditions, grafting, and chilling treatment

Cucumber (*C. sativus* L. ‘Xintai Mici’, as housekeeping homozygous material) (Csa) and Cmo (*Cucurbita moschata* ‘Qianglishi’, Shouguang Hongliang Seed Co.) (Cmo) were used as the primary scion and rootstock, respectively, for grafting. Homo-, hetero-, and opposite grafting of Csa and Cmo were done using the established hypocotyl grafting method according to a previously published description [74].

Chilling treatment was performed as described in a previous study [29]. Briefly, at 7 days after grafting, around 100 seedlings of each graft combination were transferred into a light incubator under a chilling stress condition (4°C). After different periods of chilling [0 hours (control), 6 hours, or 1, 3, 5, or 6 days], entire first true leaves with petiole and whole root from 10 grafts with similar growth from each graft combination were harvested for further analysis of chlorophyll a, chlorophyll b, and carotenoid contents and relative electrolyte leakage, as well as RNA extraction.

### Quantitative analysis of chlorophyll a, chlorophyll b, carotenoid contents, relative electrolyte permeability, and malondialdehyde content

The contents of chlorophyll a, chlorophyll b, carotenoids, and MDA, and REP, were determined referring to previous study [29]. Three to five individual seedlings and 7–10 leaf disks per leaf for each graft combination were harvested into three replicate pools at each chilling treatment time point for further analysis.

### Principal components analysis

PCA for physiological parameters, including chlorophyll a, chlorophyll b, and carotenoid contents, was performed for samples of each graft combination from each chilling treatment time point, using the Dynamic PCA online tool (Omicshare, http://www.omicshare.com/tools/home/report/reportpca.html). The scores for each parameter obtained at different time points and the distribution of each graft combination in PC1 and PC2 are shown in Supplementary Data Table S1.

### RNA extraction, RNA library construction, and sequencing

The approaches used for RNA extraction, RNA library construction, and sequencing were as described in previous reports [29, 48]. Either after 6 hours of chilling or at the same stage in non-chilled control plants, the whole first true leaf with petiole and the whole root from all grafts were harvested for RNA extraction and deep sequencing. At least 10 seedlings of all grafting combinations from each time point were pooled into three replicates. The original data set was deposited in the NCBI Small Read Archive (accession numbers PRJNA552914 and PRJNA673087).

### Identification of mobile mRNAs by RNA-seq and RT–PCR

Based on the nonsense consensus genomes of Csa (‘Xintaimici’) and Cmo (‘Qianglishi’), the detailed strategy of mobile mRNA calling was performed as previously described [48]. Lists of transmissible mRNAs (mRNAs for which there was evidence of transmission across the heterograft boundaries) were generated for both no chilling and 6 hours of chilling conditions using the Tophat v2.2 and BLASTn programs. Verification of mRNA mobility by RT–PCR was performed using established technology [48]. Primers used in this study are listed in Supplementary Data Table S17.

### RT-quantitative PCR

Reverse transcription–quantitative PCR (RT–qPCR) was performed according to a previous study [48] with at least three technical replicates, each replicate including three to five individual plants.

### Metabolite extraction, measurement, and analysis

Either after chilling treatment or at the same stage in non-chilled control plants, the whole first true leaves with petioles and whole roots from all grafts were harvested. Four to six biological replicates (where one grafted plant was regarded as one replicate) of all grafting combinations from each time point were sampled and stored in a −80°C freezer. Frozen spouts of all samples (50 mg per repetition) were ground and suspended in a 0.125% solution of formic acid in methanol (1000 μl) at 4°C for 30 min, and sonicated for 30 min at 4°C. The supernatant was centrifuged at 12 000 *g* for 10 min at 4°C and used for metabolomic analysis.

Metabolite profiling was conducted using a UPLC system (ACQUITY UPLC; Waters, Milford, MA, USA) and hybrid quadrupole time-of-flight (Q-TOF) tandem mass spectrometry (triple-TOF-MS/MS; Triple TOF 6545 system) was performed by IGENECODE Co. (Beijing,China) according to a previously described method [90, 91] with modifications.

### Statistical analysis

#### Differentially expressed genes, Kyoto Encyclopedia of Genes and Genomes pathway and Venn analysis

The abundance of each transcript was normalized and calculated via the fragments per kilobases per million reads (FPKM) method [29, 48] with modifications. Briefly, we calculated the corresponding numbers of DEGs from the FPKM values as follows. (i) The difference in transcript abundance in the same tissue between heterologous and homologous grafts without chilling, i.e. fold difference in FPKM value (control Csa/Cmo leaf − control Csa/Csa leaf), was recorded as *Δ*1. (ii) The difference in transcript abundance in the same tissue between heterologous and homologous grafts in the 6 hours of chilling condition, e.g. fold difference in FPKM value (chilling Csa/Cmo leaf − chilling Csa/Csa leaf), was recorded as *Δ*2. (iii) The fold change in transcript abundance between chilling and control conditions, e.g. fold change in FPKM value (*Δ*2 − *Δ*1), was recorded as the Csa DEGs in the leaf. (iv) Genes with differences in transcript abundance with log_2_(fold change) >2 or log_2_(fold change) < −2 were identified as up- or downregulated DEGs, respectively. KEGG pathway and Venn analysis were both performed as described in previous studies [29, 48].

#### Hierarchical clustering analysis and metabolite normalization

In total, 722 metabolites of leaves and roots in four grafted combinations grown under control and 6 hours of chilling stress conditions were clustered. The fold changes in metabolite abundance were determined as follows. (i) The ratio of the ion intensity of each metabolite in each tissue in the 6 hours of chilling and control groups, e.g. chilling Csa/Cmo leaf/chilling Csa/Cmo leaf, was calculated and recorded as *R*. (ii) The log_2_ of *R* was taken and the resulting log_2_(*R*) value was recorded as *Z*. (iii) The *Z* value of each metabolite was assessed, and the metabolites with *Z*_min_ > 2 or *Z*_max_ < −2 were retained for analysis. Following these calculations, 542 metabolites were ultimately used for further cluster analysis. The clustering was conducted using the heat map from Omicshare online tools (http://www.omicshare.com/tools) with clustering.

The metabolite abundance values were normalized using the min–max normalization method as follows:

$$ {x}^{\ast }=\frac{x-\min }{\max -\min } $$

where *x* is the original intensity, x^*^ is the normalized intensity of a given metabolite, and the min and max values are the minimum and maximum intensities for the eight metabolites from all samples. Graphs were drawn using GraphPad Prism 5.0.

#### Differentially intense metabolite analysis

The corresponding DIMs, based on the ion intensity were analyzed as follows. (i) The difference in ion intensity values between the same tissues of heterografts and homografts in the control condition, i.e. the fold difference in ion intensity (control Csa/Cmo leaf − control Csa/Csa leaf), was recorded as *Δ*1. (ii) The difference in ion intensity values between the same tissues in heterografts and homografts in the 6 hours of chilling condition, e.g. the fold change in ion intensity (chilling Csa/Cmo leaf − chilling Csa/Csa leaf), was recorded as *Δ*2. (iii) The final difference between the values from chilling and control conditions, e.g. the fold change in ion intensity in (*Δ*2 − *Δ*1), was recorded as the multiple fold change of heterologous grafts in the chilling condition. (iv) The value of log_2_(*Δ*2 − *Δ*1) was used to verify DIM status: compounds with log_2_(*Δ*2 − *Δ*1) >2 or log_2_(*Δ*2 − *Δ*1) < −2 were identified as up- or downregulated DIMs, respectively.

### Integrative analysis of metabolites, transcripts, mobile mRNA, and metabolic pathways

Pearson correlation coefficients were calculated for metabolome and transcriptome data integration. The mean of all biological replicates of each cultivar in the metabolome data and the mean value for the expression of each transcript in the transcriptome data were calculated. For example, the fold differences in Cmo/Csa root tissues were calculated from both the metabolome and the transcriptome data and compared with those of the control Csa/Csa root under both control and 6 hours of chilling conditions. The coefficients were calculated from log_2_(fold change) of each metabolite and log_2_(fold change) of each transcript using Excel software.

The relationship between metabolome, transcriptome, and mobile mRNAs was determined based on KEGG map IDs using the Excel VLOOKUP function. Then, the circlize R package was used to process the data related to metabolites and the corresponding mobile mRNAs showing changes in expression were used to draw chord (Circos) diagrams in RStudio [92]. We used Circos diagrams to analyze the correlation between the rootstock/scion of each heterologous grafting combination. The Pearson correlation was calculated using the corrplot R package [93] between metabolites and the corresponding mobile mRNA. Metabolome, transcriptome, and mobile mRNA relationships were visualized using Cytoscape (version 3.8.1) [94].

### Exogenous application of metabolite solutions

The exogenous solutions included PE (CAS#39382-08-6, soluble in chloroform; Aladdin Biochemical Technology Co., Shanghai, China), PC, lecithin, (CAS#8002-43-5, soluble in ethanol; Aladdin Biochemical Technology Co., Shanghai, China), phosphorylcholine chloride (PCho, CAS#107-73-3, soluble in water; Macklin Biochemical Technology Co., Shanghai, China), *O*-phosphorylethanolamine (PEth, CAS#1071-23-4, soluble in water; Aladdin Biochemical Technology Co., Shanghai, China), choline (CAS#62-49-7, soluble in water; Macklin Biochemical Technology Co., Shanghai, China) and Eth (CAS#141-43-5, soluble in water; Aladdin Biochemical Technology Co., Shanghai, China), with a concentration gradient (0, 20, 40, 60, 80, 100 μmol/l). The metabolite solutions were supplied to leaves of two-leaf-stage Csa seedlings every morning and evening for 3 days before chilling stress. PE was dissolved in chloroform (v:v 0.1% in water). Normal water and blank solution were both used as control. Then, chilling treatment was performed at 4°C at different time points. At least three replicate pools, each pool including 9 or 10 individual plants, were included in both the control and the treatment group. The mixture solution of PCho and PEth at different concentrations (μmol/l) included PCho: PEth 80:0, 80:40, 80:80. PCho and PEth were dissolved in 0.1% ethanol and 0.1% chloroform. Therefore, control 1 was a 2:1 (v:v) mixture solution of 0.1% ethanol and 0.1% chloroform with 0 μM PCho and 0 μM PE. Control 2 was a solution of 0.1% chloroform with 0 μM PEth.

## Acknowledgements

We greatly thank Beijing IgeneCode Biotech Co., Ltd (www.igenecode.com) for support in metabolome measurement and analysis of the original dataset. This work was supported by grants from the National Key Research and Development Program of China (2018YFD1000800 and 2019YFD1000300) to WN Zhang, the National Natural Science Foundation of China (31872158) to WN Zhang, Fundamental Research Funds for ‘Talent Cultivation and Development Plan’ of China Agricultural University (00109016), and China Agriculture Research System of MOF and MARA to LH Gao.

## Author contributions

L.H.G. and W.N.Z. conceived and designed the experiments; W.Q.L., Q.W., X.J.L., T.W., X.H.L., Z.X.L., R.Y.Z., C.C.W., and M.S.L. performed the experiments; W.Q.L., X.J.Z., and W.N.Z. wrote the paper; W.Q.L., C.G.X., Q.W., and W.N.Z. analyzed the data; all authors read the final version of this manuscript and approved it for publication.

## Data availability

The original data set was deposited in the NCBI Small Read Archive (accession numbers PRJNA552914 and PRJNA673087). Other data supporting the results of this article are included within the article and its additional files.

## Conflict of interest

None declared.

## Supplementary data

Supplementary data is available at *Horticulture Research* online.

## Supplementary Material

Web_Material_uhac031
