# Supplementary material for: Rootstock–scion exchanging mRNAs participate in the pathways of amino acid and fatty acid metabolism in cucumber under early chilling stress
Source: Hortic Res. 2022 Feb 19;9:uhac031. doi: 10.1093/hr/uhac031 (PMC9039506; doi:10.1093/hr/uhac031)

Supplementary Material

Supplementary figures legends

Supplementary FIG. S1. Changes in the relative expression of cold pathway genes in grafted combinations after 6h chilling stress. *CmoACTIN7* or *CsaACTIN7* was used as an internal reference for Cmo or Csa samples. Specific primers recognizing Cmo or Csa transcripts were used for RT-qPCR. Graphs were generated by GraphPad Prism5.0. Black asterisk indicated significant differences (using t-test; p<0.05,*; p<0.01,**; p<0.001,***) between Cmo or Csa tissues in grafted combinations after chilling treatment for 6h. Csa, *Cucumis sativus*; Cmo, *Cucurbita moschata*.

Supplementary FIG. S2. PCA of metabolite profiles in leaf and root of all grafted combinations under control and 6 h chilling stress conditions without filtration. 30 samples from the first leaves of scions and 27 samples from roots of rootstocks after chilling stress and in the normal condition were included in PCA analysis, which showed a clear separation between different tissues and species

Four independent biological replicates (one grafted plant was regarded as one replicate) of all grafting combinations from each treatment group (control and 6 h chilling) were analyzed. Values were $\log_2$-median-transformed averages.

Supplementary FIG. S3. Hierarchical cluster analysis (HCA) of 542 metabolites from leaves and root of all graft combinations under chilling conditions. (a). Heatmap of $\log_2$-median transformed average fold change (6 h chilling/no-chilling control) of four independent leaves and roots of grafts in no-chilling control and 6 h chilling stress conditions. The cluster was conducted by using Heatmap with cluster in Omicshare online tools (http://www.omicshare.com/tools). Red asterisks indicated examples of a significant 6h/0h foldchange of the respective metabolites in different tissues. (b). Normalized responses of 8 metabolites in clusters I–VIII. Detailed value of each comparison (6 h chilling/no-chilling control) are provided in Supplementary Table S4.

Supplementary FIG. S4. Analysis of significantly differentially expressed genes between *C. sativus* and *C. moschata* under chilling stress. (a) Upregulated and downregulated DEGs number of *C. sativus* and *C. moschata* under chilling stress. (b and c) Violin diagram of all DEGs in Cmo and Csa tissues after chilling treatment for 6 h.

Supplementary FIG. S5. Integrated analysis between chilling-induced mobility of mRNAs and DEGs. Venn diagram of 152 chilling-induced mobile Cmo mRNAs (a) and 148 homologous Csa mRNAs (d) compared with Cmo differentially expressed genes (DEGs). (b).Venn diagram of 107 chilling-induced mobile Cmo mRNAs with Cmo DEGs and 95 chilling-induced mobile Cmo mRNAs with Csa DEGs. Venn diagram of 1,750 chilling-induced mobile Csa mRNAs (c) and 1,718 homology Cmo mRNAs (f) compared with Cmo DEGs (e). Venn diagram of 877 chilling-induced mobile Csa mRNAs with Csa DEGs and 772 chilling-induced mobile Csa mRNAs with Cmo DEGs change. KEGG pathway analysis of 49 chilling-induced mobile Cmo mRNAs with DEGs (g) and 481 chilling-induced mobile Csa mRNAs with DEGs (h). Venn diagram and KEGG analysis are performed by the online OmicShare tools (http://www.omicshare.com/tools).

Supplementary FIG. S6. Integrated analysis between chilling-reduced mobility of mRNAs and DEGs. Venn diagram of 2173 chilling-reduced mobile Csa mRNAs (a) and 2104 homologous Cmo mRNAs (b) compared with Cmo differentially expressed genes (DEGs) change. (c).Venn diagram of 1693 chilling-reduced mobile Cmo mRNAs with Cmo DEGs change and 1496 chilling-reduced mobile Csa mRNAs with Csa DEGs change. (d).Venn diagram of 1254 chilling-reduced mobile Csa mRNAs (a) and 1442 homology Cmo mRNAs (b) compared with Cmo DEGs change. (f). Venn diagram of 1280

chilling-reduced mobile Cmo mRNAs with Cmo DEGs (c) and 1054 chilling-reduced mobile homologous Cmo mRNAs with Csa DEGs (d). KEGG pathway analysis of 949 chilling-reduced mobile Csa mRNAs associating with DEGs (g) and 628 chilling-reduced mobile Cmo mRNAs associating with DEGs (h). Venn diagram and KEGG analysis are performed by the online OmicShare tools (http://www.omicshare.com/tools).

Supplementary FIG. S7. Integrated analysis between mobility-direction changed mRNAs and DEGs. Venn diagram of 317 mobility-direction changed Csa mRNAs (a) and 312 homologous Cmo mRNAs (b) compared with Cmo differentially expressed genes (DEGs). (c).Venn diagram of 51 mobility-direction changed Cmo mRNAs with Cmo DEGs change and 51 mobility-direction changed homologous Csa mRNAs with Csa DEGs. (d).Venn diagram of 262 mobility-direction changed Csa mRNAs (a) and 192 homology Cmo mRNAs (b) compared with Cmo DEGs. (f). Venn diagram of 42 mobility-direction changed Cmo mRNAs with Cmo DEGs (c) and 41 mobility-direction changed homologous Cmo mRNAs with Csa DEGs (d). KEGG pathway analysis of 167 mobility-direction changed Csa mRNAs associating with DEGs (g) and 22 mobility-direction changed Cmo mRNAs associating with DEGs (h). Venn diagram and KEGG analysis are performed by the online OmicShare tools (http://www.omicshare.com/tools).

Supplementary FIG. S8. Integrated analysis of chilling-reduced mobile mRNAs, differentially expressed genes (DEGs) and differentially intense metabolites (DIMs)

Chord diagrams of chilling-reduced mobile Cmo mRNAs, metabolites, Csa DEGs and metabolic pathways in cucumber leaf and root of heterografts (a) and pumpkin leaf (c) created with the circlize R package. (b).Networks involving the metabolites 2-Oxoglutarate, Oxaloacetate, Acetaldehyde, S-Adenosyl-methioninamineand related Csa DEGs and chilling-reduced homologous mobile Cmo mRNAs. Blue dotted line box indicated the correlation in cucumber roots. (d). Networks involving the metabolites Succinate, Phenylpyruvate, 4-Coumarate, Phenylpyruvate, (S)-Malate and related Csa DEGs and chilling-reduced homologous mobile Cmo mRNAs. The DEGs in cucumber and pumpkin tissues of heterografts associated with at least one chilling-reduced mobile mRNAs are visualized with Cytoscape (version 3.8.1). Red circles indicate the two metabolites, blue circles indicate DEGs and orange circles indicate DEG-coordinated homologous mobile mRNAs. Arrows (red for positive and blue for negative correlation, gray for the corresponding relationship) indicate the correlation between metabolites and DEGs.

Supplementary FIG. S9. Integrated analysis of mobility direction changed and chilling-induced mobility of Csa mRNAs, differentially expressed genes (DEGs) and differentially intense metabolites (DIMs) in pumpkin

Chord diagrams of mobility direction changed Csa mRNAs (a), chilling-induced mobility of Csa mRNAs (c), DIMs, Cmo DEGs and metabolic pathways in pumpkin leaf of heterografts created with the circlize R package. (b). Networks involving the metabolites related Cmo DEGs and mobility direction changed Csa mRNAs. (d). Networks involving the metabolites and related Cmo DEGs and chilling-induced homologous mobile Csa mRNAs. The DEGs in cucumber and pumpkin tissues of heterografts associated with at least one chilling-reduced mobile mRNAs are visualized with Cytoscape (version 3.8.1). Red circles indicate the two metabolites, blue circles indicate DEGs and orange circles indicate DEG-coordinated homologous mobile mRNAs. Arrows (red for positive and blue for negative correlation, gray for the corresponding relationship) indicate the correlation between metabolites and DEGs.

Supplementary FIG. S10. RT-PCR verification of predicted mobile mRNAs.

Red arrows indicated mobile direction and red line indicated unidentified mobility. Each sample included

9-10 biological replicates and 3-5 technique replicates.

Supplementary FIG. S11. The effect of exogenous metabolites application on the chilling tolerance of cucumber seedlings

(a) Exogenous phosphatidylcholine (PC), phosphatidylethanolamine (PE), choline (Cho) and ethanolamine (Eth) application in two-leaf-stage cucumber seedling. The solution of chloroform (v:v 0.1%) was also used as control. (b), Relative electrolyte permeability (REP) of first leaves under different conditions after chilling treatment for 24 h. Six individual plants at each time points were used for analysis. Student t-test was used to analyzed the significant difference. Asterisks indicate significant difference in each time points comparing with normal (**$P < 0.001$, *$P < 0.05$).

Supplementary tables legends

Supplementary Table S1. Principal component analysis of the physiological traits of all graft combinations under chilling stress

Supplementary Table S2. Ion intensity values of 760 metabolites in leaf and root of all four cucumber and pumpkin graft combinations under control conditions and after 6 h chilling

Supplementary Table S3. Principal component analysis of all samples and the contribution of 542 metabolites in PC1 and PC2 levels

Supplementary Table S4. Clusters identified among the 542 metabolites tested in different organs of all grafted combinations under control conditions and chilling stress

Supplementary Table S5. Differentially Expressed Genes (DEGs) in leaves and roots of heterografted cucumber seedlings under chilling stress

Supplementary Table S6. Kyoto Encyclopedia of Genes and Genomes (KEGG) Pathway analysis of the different clusters of differentially expressed genes (DEGs) in cucumber tissues of heterografts under chilling stress

Supplementary Table S7. Kyoto Encyclopedia of Genes and Genomes (KEGG) Pathway analysis of the different cluster of differentially expressed genes (DEGs) in pumpkin tissues of heterografts under chilling stress

Supplementary Table S8. Mobile mRNAs in cucumber and pumpkin under control and 6 h chilling conditions

Supplementary Table S9. Mobility categories of mobile mRNAs based on how they migrate between cucumber and pumpkin under control and 6 h chilling conditions

Supplementary Table S10. Kyoto Encyclopedia of Genes and Genomes (KEGG) Pathway analysis of the different clustered mobile mRNA in cucumber and pumpkin under control and 6 h chilling conditions

Supplementary Table S11. Kyoto Encyclopedia of Genes and Genomes (KEGG) Pathway analysis of different clustered mobile mRNA correlating with Csa/CmoDEGs under control and 6 h chilling conditions

Supplementary Table S12. Correlations between DEGs and DIMs in cucumber and pumpkin leaf and root of all graft combinations

Supplementary Table S13. The correlated DEGs and DIMs filtered duplications in cucumber and pumpkin leaf and root of all graft combinations

Supplementary Table S14. Kyoto Encyclopedia of Genes and Genomes (KEGG) Pathway analysis of correlated DEGs and DIMs filtered duplications in cucumber and pumpkin leaf and root of all graft combinations

Supplementary Table S15 Correlation analysis of CsaDIMS, CsaDEGs and chilling-induced mobile Cmo RNAs in cucumber tissues of heterografts in responding to chilling

Supplementary Table S16 Key enzymes and corresponding genes in the KEGG pathway of amino acid biosynthesis and fatty acid metabolism

Supplementary Table S17 Oligonucleotides of RT-qPCR used in the study

Supplementary Figure S1

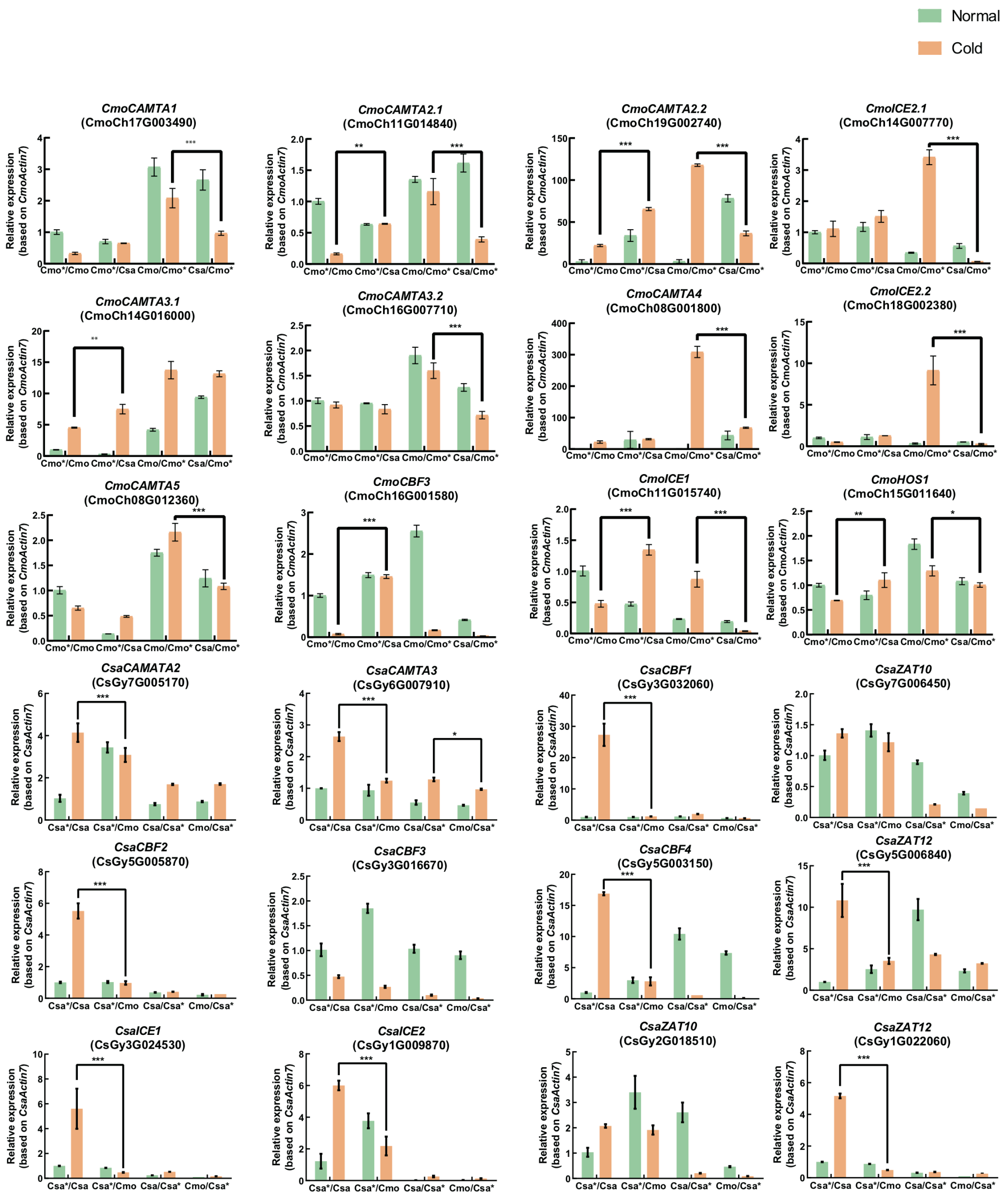
Normal
Cold
CmoCAMTA1 (CmoCh17G003490)
CmoCAMTA2.1 (CmoCh11G014840)
CmoCAMTA2.2 (CmoCh19G002740)
CmoICE2.1 (CmoCh14G007770)
CmoCAMTA3.1 (CmoCh14G016000)
CmoCAMTA3.2 (CmoCh16G007710)
CmoCAMTA4 (CmoCh08G001800)
CmoICE2.2 (CmoCh18G002380)
CmoCAMTA5 (CmoCh08G012360)
CmoCBF3 (CmoCh16G001580)
CmoICE1 (CmoCh11G015740)
CmoHOS1 (CmoCh15G011640)
CsaCAMATA2 (CsGy7G005170)
CsaCAMTA3 (CsGy6G007910)
CsaCBF1 (CsGy3G032060)
CsaZAT10 (CsGy7G006450)
CsaCBF2 (CsGy5G005870)
CsaCBF3 (CsGy3G016670)
CsaCBF4 (CsGy5G003150)
CsaZAT12 (CsGy5G006840)
CsaICE1 (CsGy3G024530)
CsaICE2 (CsGy1G009870)
CsaZAT10 (CsGy2G018510)
CsaZAT12 (CsGy1G022060)
Relative expression (based on CmoActin7)
Relative expression (based on CsaActin7)
Cmo*/Cmo
Cmo*/Csa
Cmo/Cmo*
Csa/Cmo*
Csa*/Csa
Csa*/Cmo
Csa/Csa*
Cmo/Csa*

Supplementary Figure 2

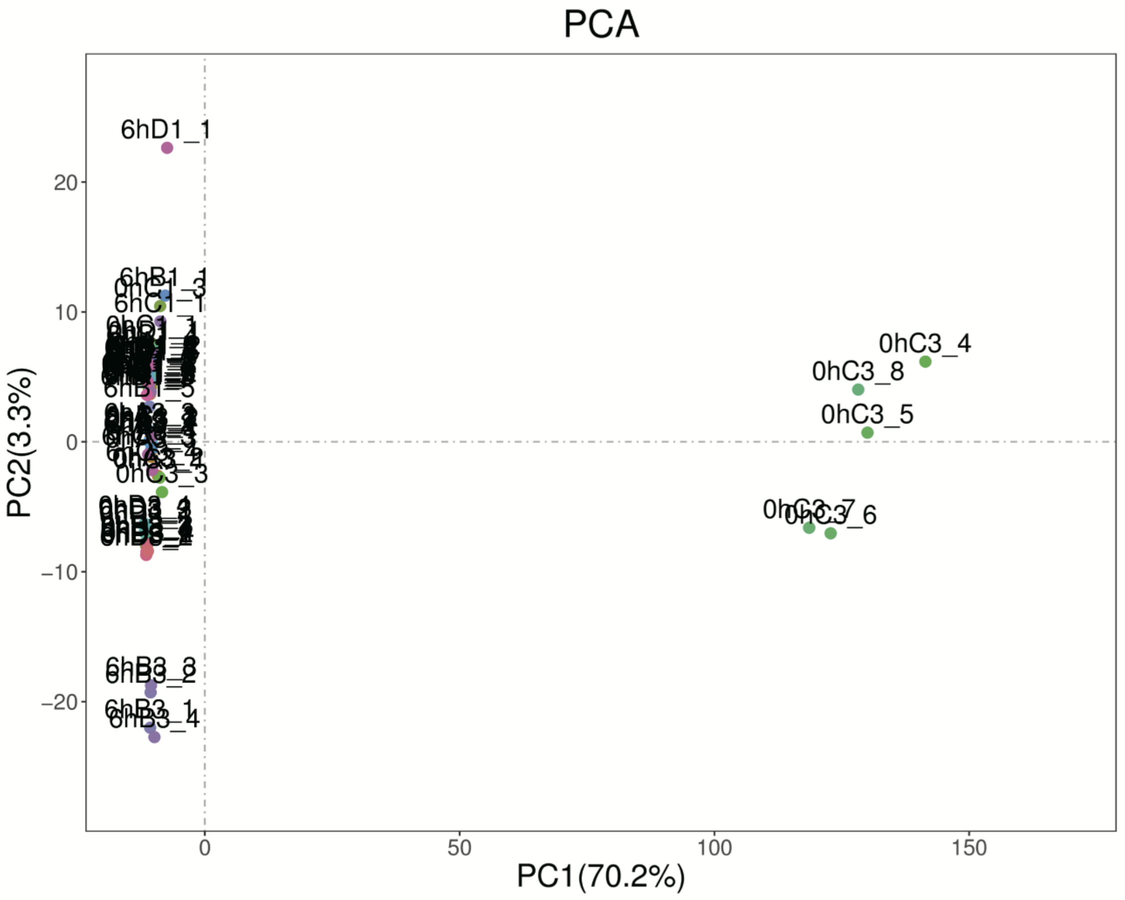

Supplementary figure S3

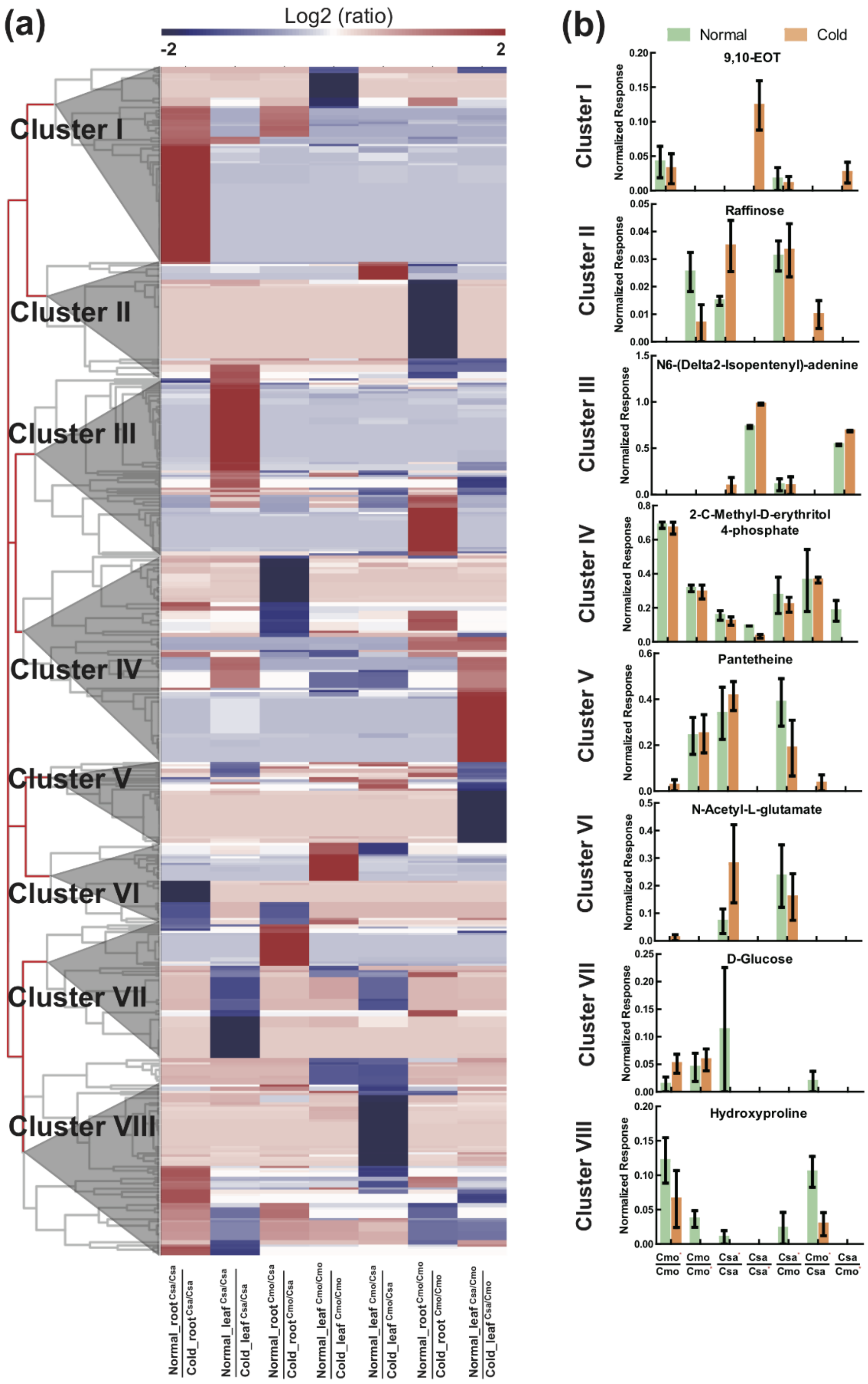

Supplementary Figure 4

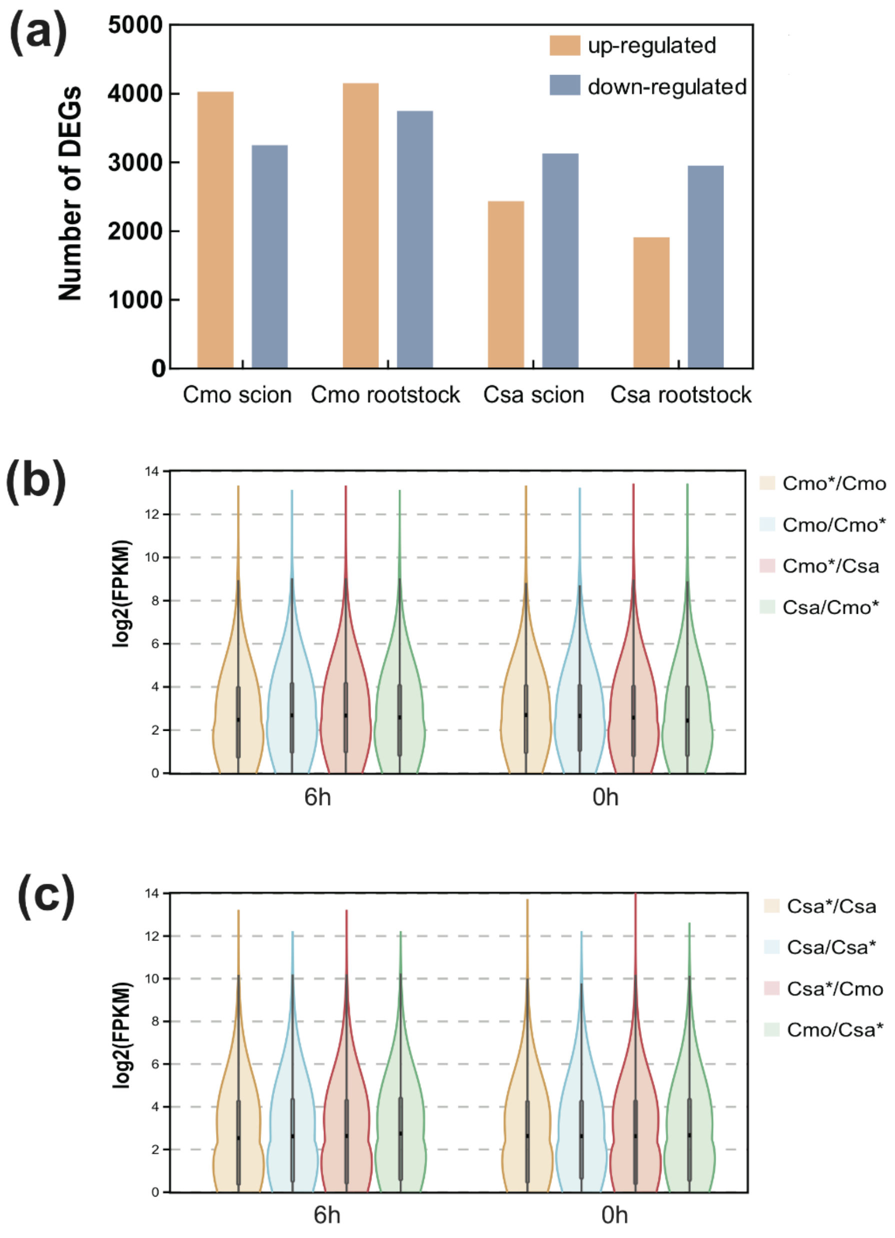

Supplementary Figure 5

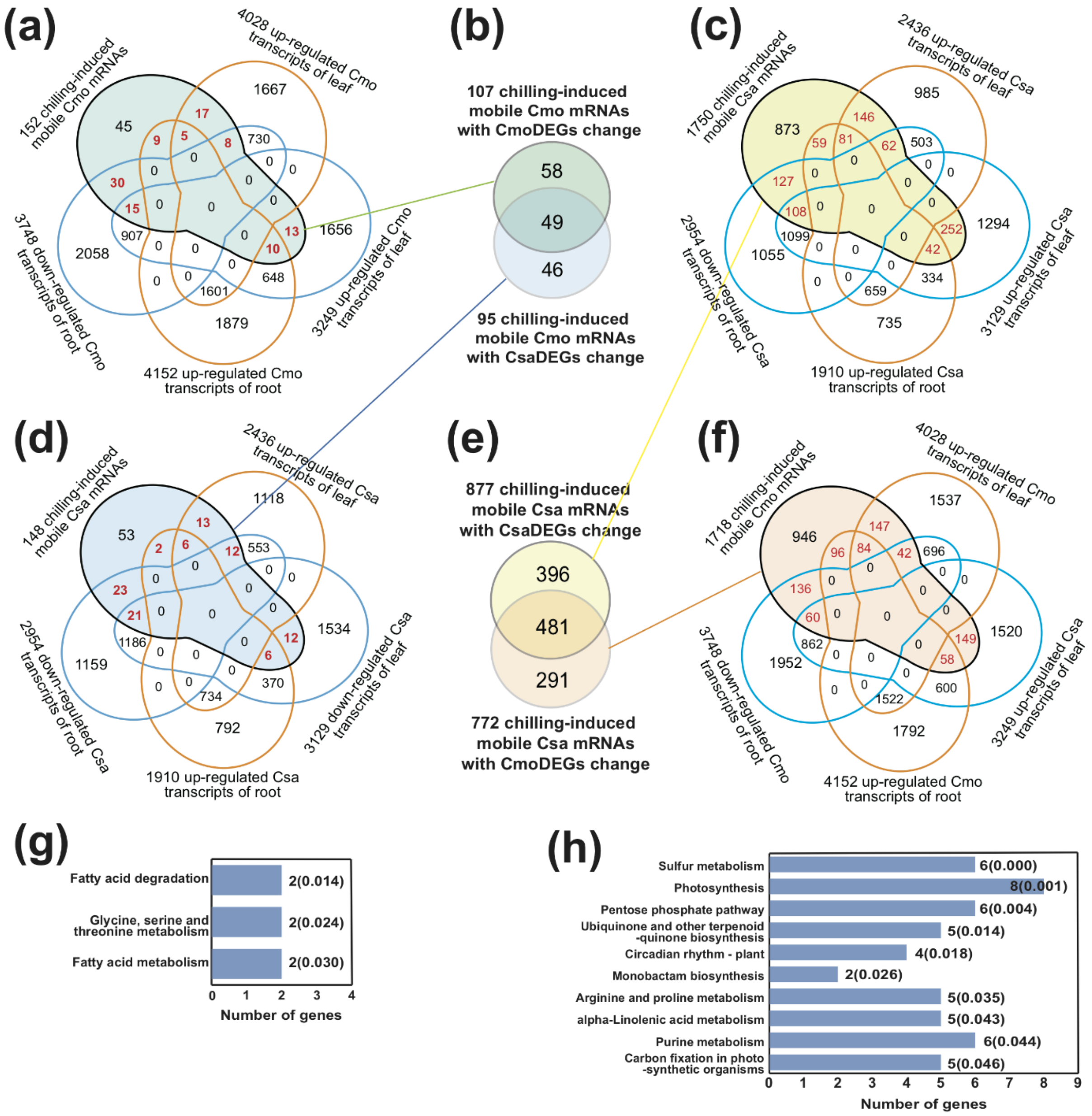

Supplementary Figure 6

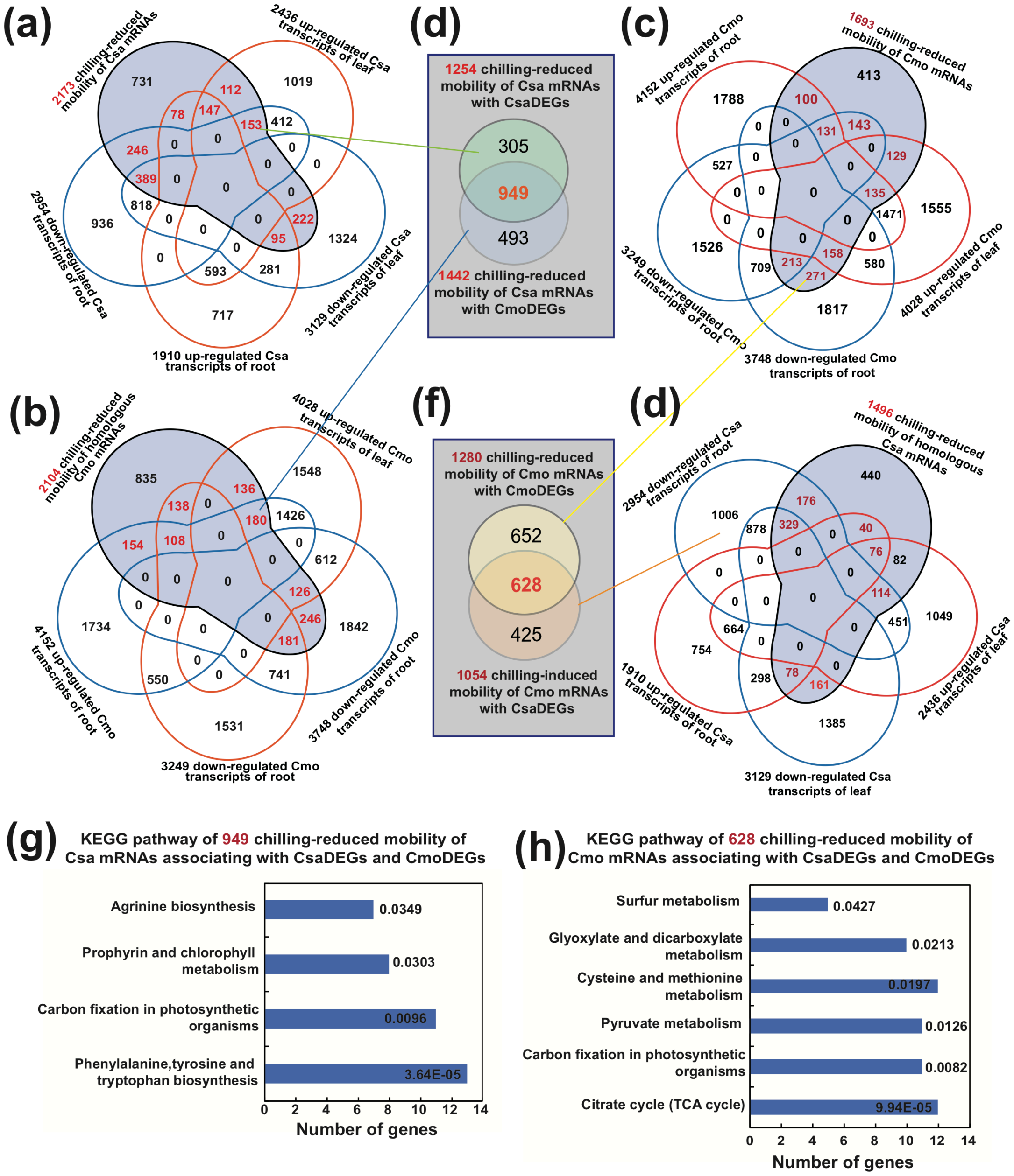

Supplementary Figure 7

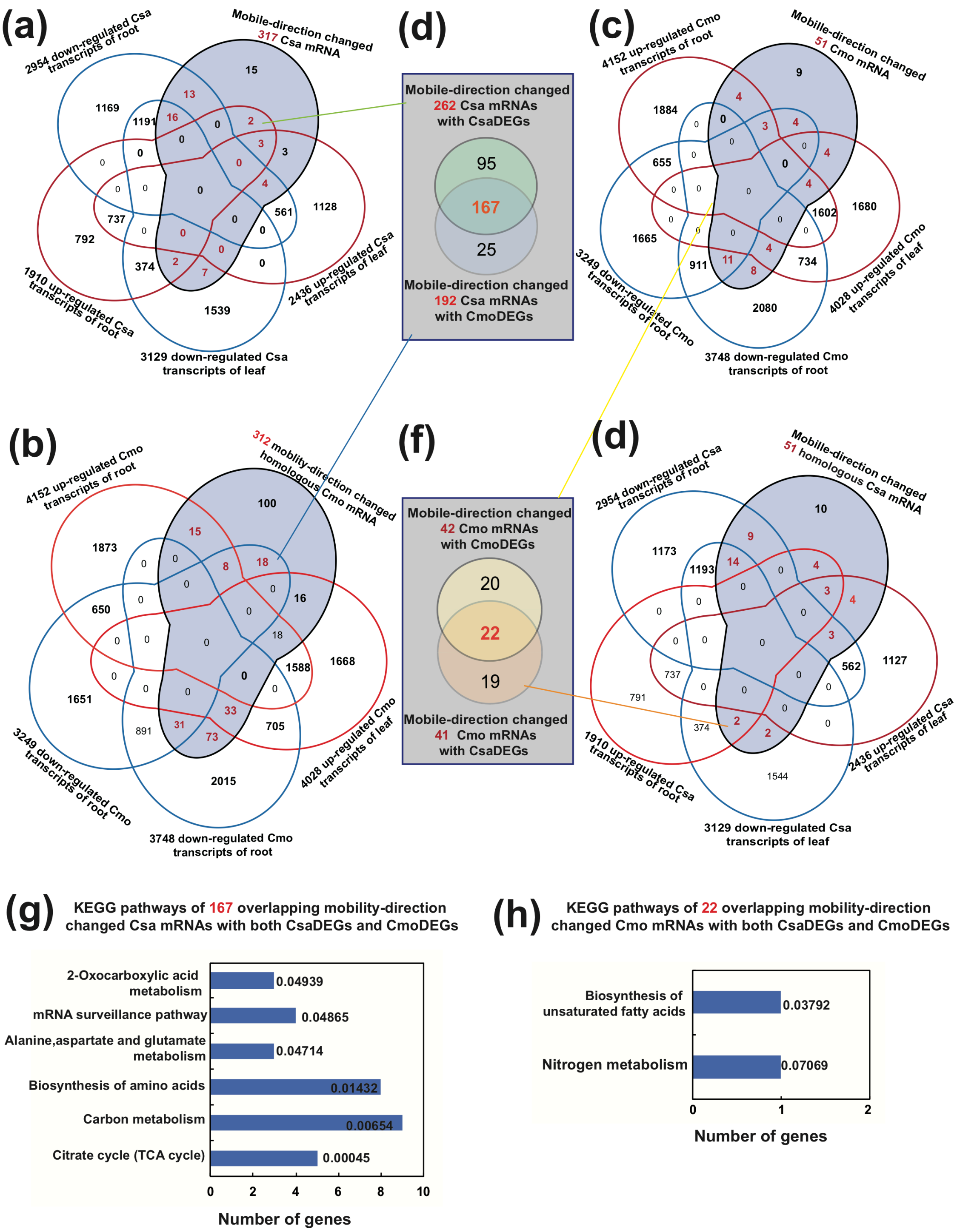

Supplementary Figure 8

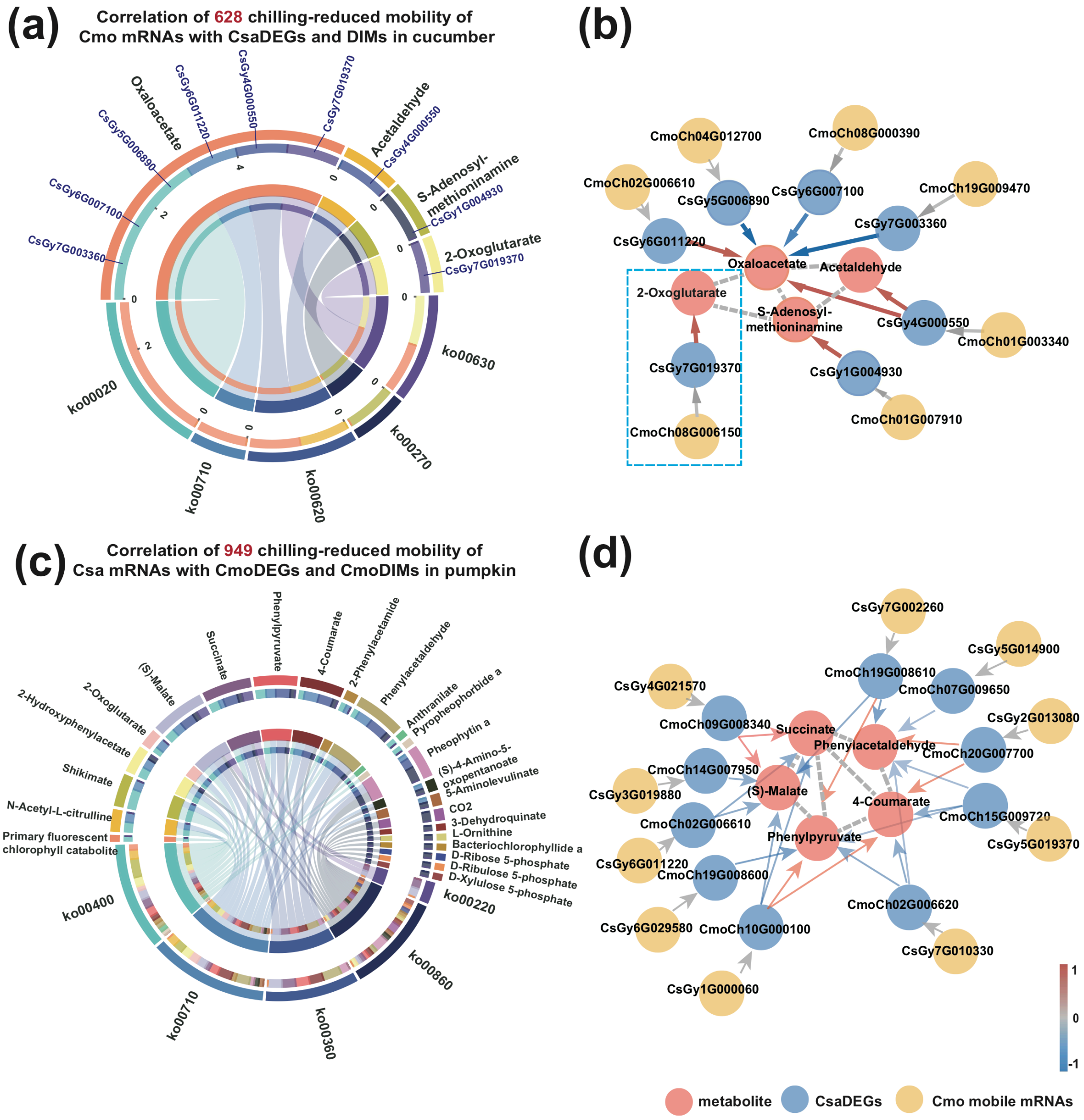

Supplementary Figure 9

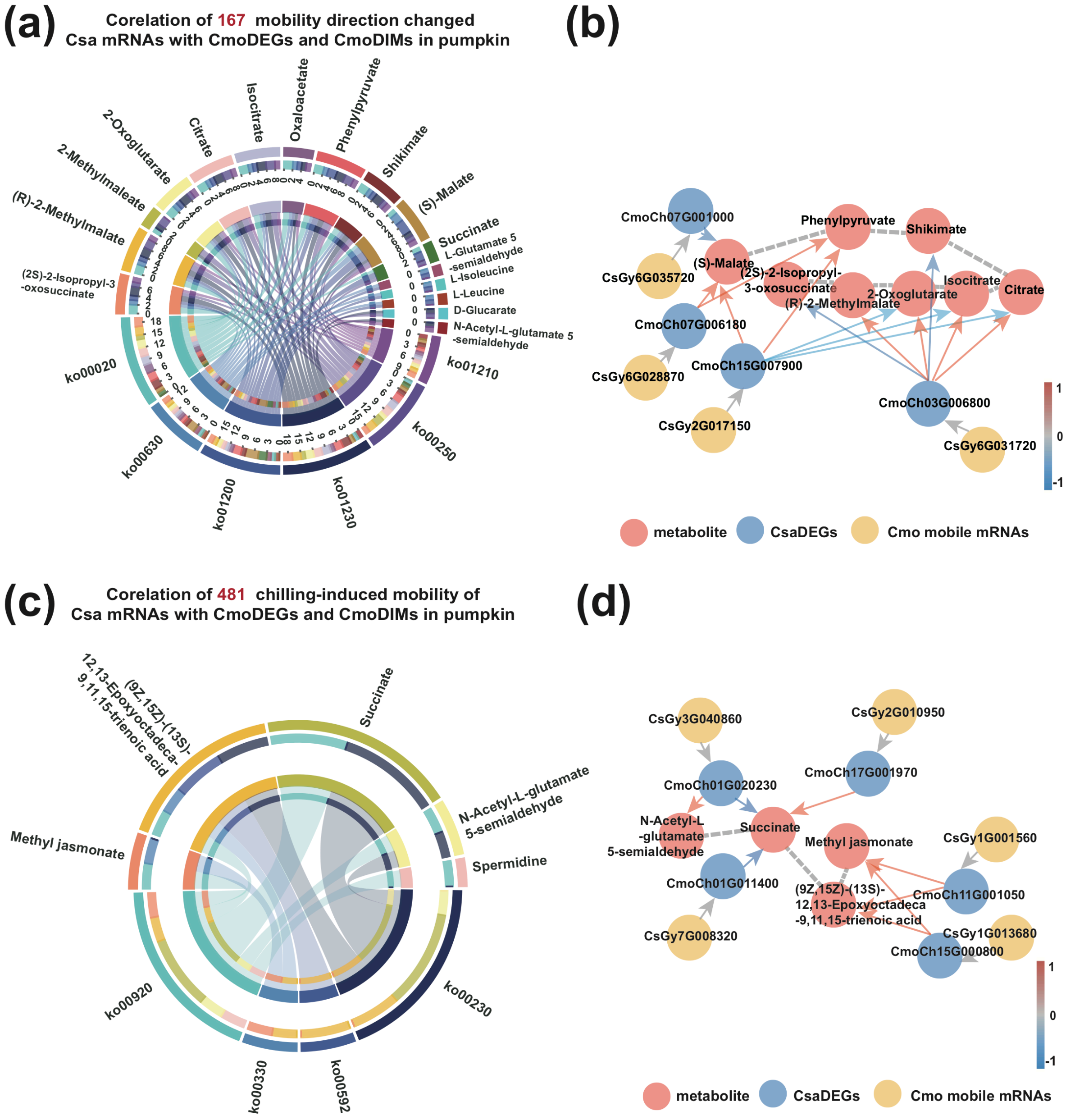

Figure S10

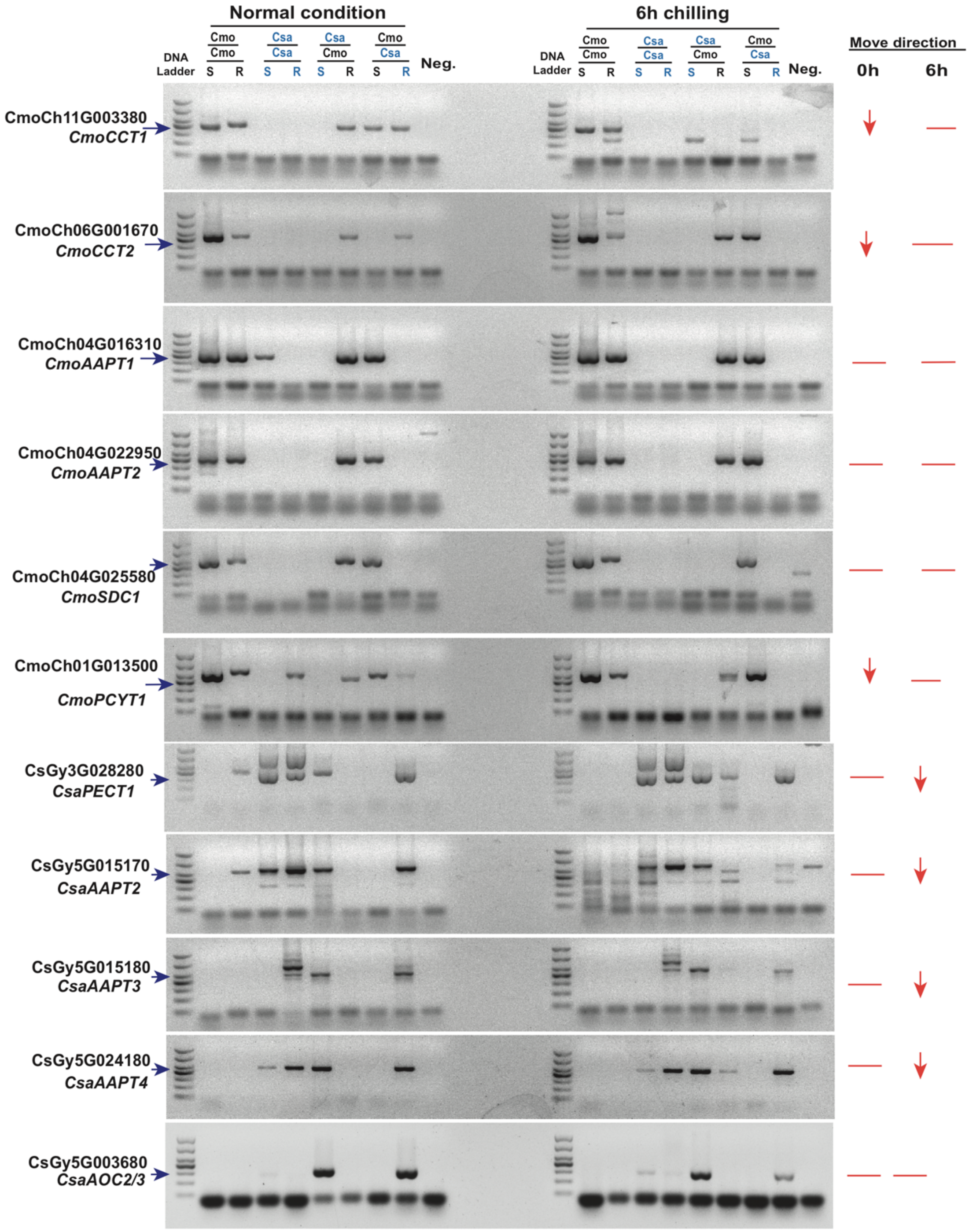
Normal condition
6h chilling
DNA Ladder
Cmo/Cmo
Csa/Csa
Csa/Cmo
Cmo/Csa
S R S R S R S R
Neg.
Move direction
0h
6h
CmoCh11G003380
CmoCCT1
CmoCh06G001670
CmoCCT2
CmoCh04G016310
CmoAAPT1
CmoCh04G022950
CmoAAPT2
CmoCh04G025580
CmoSDC1
CmoCh01G013500
CmoPCYT1
CsGy3G028280
CsaPECT1
CsGy5G015170
CsaAAPT2
CsGy5G015180
CsaAAPT3
CsGy5G024180
CsaAAPT4
CsGy5G003680
CsaAOC2/3

Supplementary Figure 11

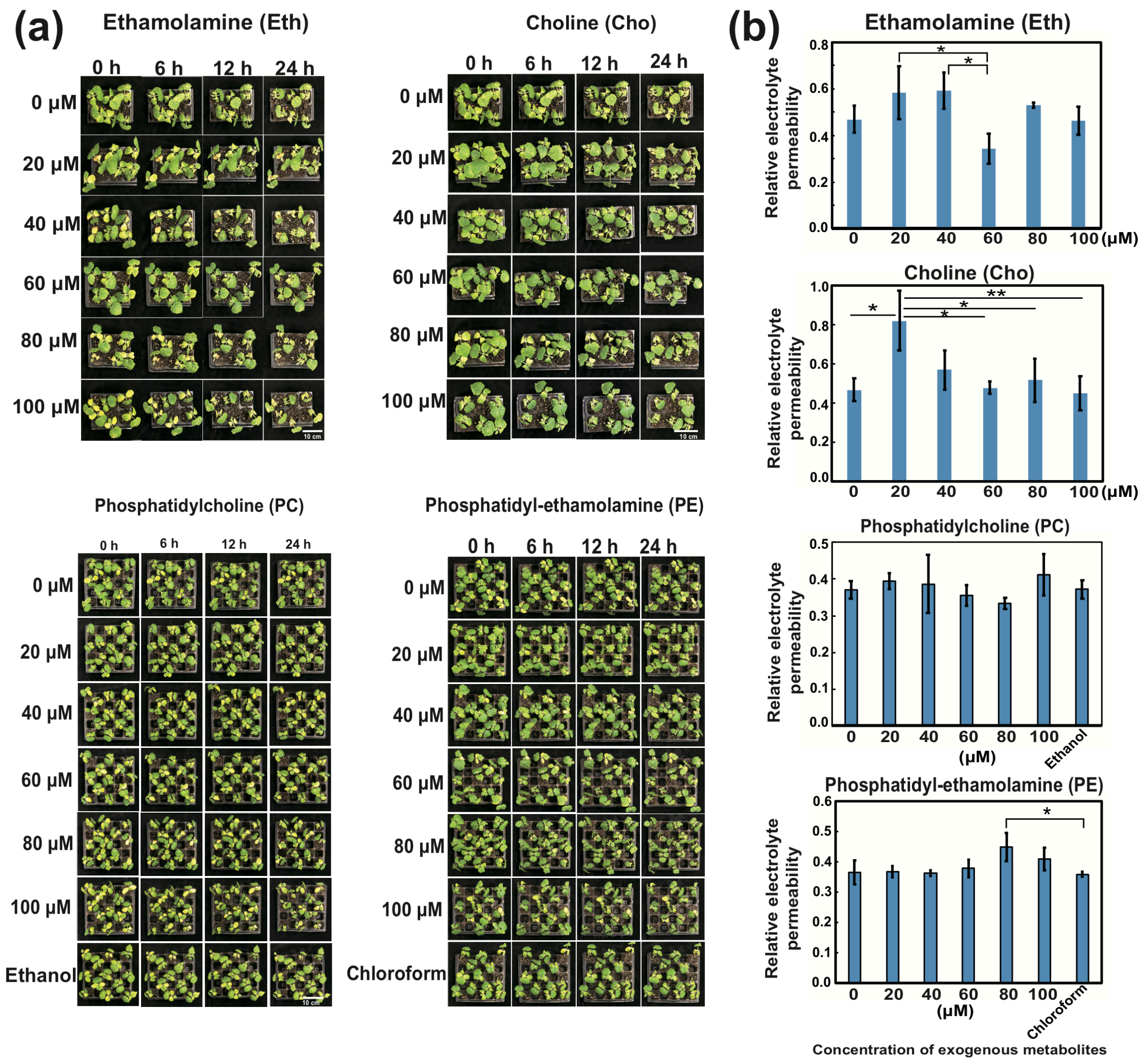

Supplement: Web_Material_uhac031 [file web_material_uhac031.zip › Supplementary metarial.pdf]
